# Changes in pulmonary artery size during and after staged extracardiac total cavopulmonary connection

**DOI:** 10.1016/j.xjon.2026.101721

**Published:** 2026-03-12

**Authors:** Teresa Lemmen, Thibault Schaeffer, Christina Ruda, Adrian Lehmann, Jonas Palm, Nicole Piber, Muneaki Matsubara, Paul Philipp Heinisch, Stanimir Georgiev, Alfred Hager, Peter Ewert, Jürgen Hörer, Masamichi Ono

**Affiliations:** a Department of Congenital and Pediatric Heart Surgery, TUM University Hospital, German Heart Center, Munich, Germany; b Division of Congenital and Pediatric Heart Surgery, University Hospital of Munich, Ludwig-Maximilians-Universität München, Munich, Germany; c Europäisches Kinderherzzentrum München, Munich, Germany; d Department of Congenital Heart Disease and Pediatric Cardiology, TUM University Hospital, German Heart Center, Munich, Germany; e Department of Cardiovascular Surgery, TUM University Hospital, German Heart Center, Munich, Germany

**Keywords:** total cavopulmonary connection, pulmonary artery size, symmetry index

## Abstract

**Background:**

Optimal development of the pulmonary arteries (PAs) is crucial for Fontan circulation. This study investigated longitudinal changes in PA size in patients who underwent staged Fontan palliation and analyzed their impact on outcomes.

**Methods:**

Serial PA angiograms at bidirectional cavopulmonary shunt (BCPS), extracardiac total cavopulmonary connection (TCPC), and after TCPC were reviewed. PA index (PAI), right and left PAI, and PA symmetry index were calculated, and serial changes were compared.

**Results:**

Among 391 patients, right BCPS was performed in 328 (83.9%). The median age at TCPC was 2.1 years (interquartile range: 1.7-2.6 years). The most frequent diagnosis was hypoplastic left heart syndrome, in 148 patients (37.9%). PAI did not change between BCPS and TCPC (*P* = .564) or between pre-TCPC and post-TCPC (*P* = .569); however, right PAI increased between BCPS and TCPC (*P* = .007), while left PAI decreased (*P* < .001). Neither right PAI (*P* = .341) nor left PAI (*P* = .413) changed significantly between pre-TCPC and post-TCPC. The PA symmetry index decreased between BCPS and TCPC (*P* < .001) but remained stable thereafter (*P* = .912). Pre-TCPC small PAI predicted prolonged pleural effusion (*P* = .009; odds ratio [OR], 0.995) and ascites (*P* = .005; OR, 0.990). Pre-TCPC low symmetry index was a risk factor for mortality (*P* = .032; hazard ratio, 12.930), and its cutoff of 0.61 discriminated transplantation-free survival (*P* = .006).

**Conclusions:**

During the staged Fontan procedure, the left PAI decreased, whereas the PAI and right PAI remained stable or increased. A PA symmetry index <0.61 predicts mortality following TCPC. This progressive asymmetry in left-to-right PA size affects in-hospital morbidity and emphasizes the importance of achieving balanced PA growth prior to Fontan completion.

## Graphical Abstract

**Figure:**
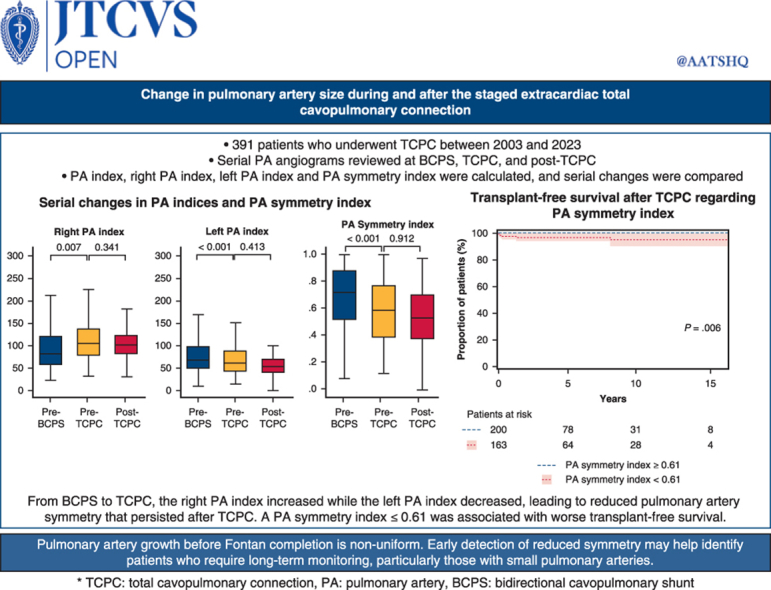

**Figure:**
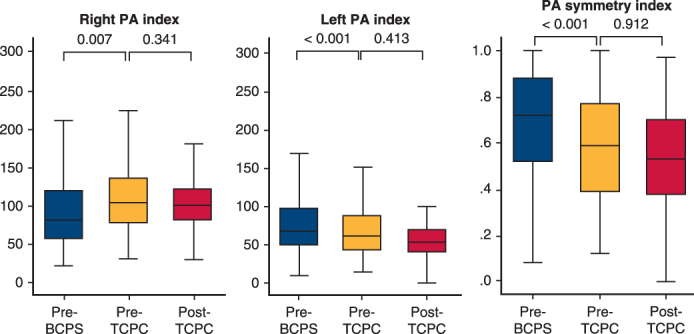
Serial changes in pulmonary artery indices and the pulmonary artery symmetry index.

**Central Message**

Pulmonary artery growth becomes unbalanced between bidirectional cavopulmonary shunt and total cavopulmonary connection, with the right pulmonary artery index (PAI) increasing and the left PAI decreasing, resulting in reduced symmetry without further significant change in the indices.

**Perspective**

Unbalanced pulmonary artery growth before Fontan completion results in persistent right-sided dominance of pulmonary blood flow. This leads to a higher risk for morbidities after the surgeries, highlighting the importance of early recognition and long-term monitoring of patients with asymmetric and small pulmonary arteries.

The Fontan procedure creates passive pulmonary blood flow by directing systemic venous return directly into the pulmonary arteries (PAs).^1^ After several modifications, the current preferred strategy is staged Fontan completion through a bidirectional cavopulmonary shunt (BCPS), followed by a total cavopulmonary connection (TCPC).^2^, ^3^, ^4^ Once BCPS is performed, the circulation depends entirely on low-resistance passive PA flow, making PA size a critical factor.^5^

Well-balanced PA development is essential, as small or unbalanced PAs are associated with worse outcomes and worse candidacy for Fontan.^6^, ^7^, ^8^ Studies have shown limited changes in indexed PA size between BCPS and TCPC,^9^^,^^10^ with PA growth after TCPC often disproportionate to somatic growth.^11^^,^^12^ Small PA size following TCPC has been linked to worse functional clinical status,^13^ increased rates of chylothorax,^7^ and worse hemodynamic variables.^8^ The lower limit of acceptable PA size at Fontan completion remains controversial.^6^, ^7^, ^8^^,^^14^, ^15^, ^16^

Numerous previous studies have provided pieces of the puzzle that illustrate the adaptation to Fontan physiology, but few have investigated the longitudinal changes in PA size and growth over time.^17^, ^18^, ^19^ Furthermore, anatomic variations of the superior vena cava (SVC), such as bilateral or left-sided SVC, may influence PA development but remain unexplored. Given that PA size is a determinant factor for successful TCPC and long-term clinical outcomes, a better understanding of its changes over time is crucial. Therefore, this study aimed to provide a longitudinal assessment of PA size in patients undergoing staged Fontan palliation and to analyze its effects on clinical outcomes.

## Methods

### Data Availability Statement

The data that support the findings of this study are available from the corresponding author on reasonable request.

### Ethical Statement

This study was approved by the Institutional Review Board of the Technical University of Munich (approval 2025-232-S-NP; approved May 5, 2025). Owing to the study’s retrospective nature, the requirement for individual patient consent was waived.

### Patients and Data Collection

We reviewed all patients who underwent staged TCPC at our center between 2003 and 2023 and had pre-BCPS and/or pre-TCPC angiograms available for review. Patients without angiographic data or current clinical follow-up were excluded. Cardiac catheterization was performed routinely before BCPS and TCPC. Following TCPC, cardiac catheterization was indicated only in patients exhibiting symptoms, such as hypoxemia, or when hemodynamic evaluation was necessary. Archived PA angiograms were used for the measurement of PA diameters. The PA index (PAI) was calculated using angiography, as described by Nakata and colleagues.^20^ The diameters at the bifurcation branch of the upper and lower lobes in the right and left PA were measured from the anteroposterior view of the pulmonary angiogram. Right and left PAIs were calculated by dividing the cross-sectional area of each branch by the body surface area. The PA symmetry index was calculated as described by Glatz and colleagues,^21^ and the ratio of the left PAI to the right PAI was calculated. Serial changes in PAIs were compared in all patients and in patients with single right BCPS and other forms of BCPS. Medical records, including baseline morphology, demographics, and preoperative, intraoperative, and postoperative data, were reviewed. Follow-up data from the time of TCPC to the last available record were retrieved from our institutional single ventricle database. In the survival analysis, patients alive at the most recent clinical assessment were censored at that date. Death was treated as the event of interest.

### Surgical Techniques

BCPS was performed under cardiopulmonary bypass in most patients as described previously.^22^ Cardioplegic arrest was used only when intracardiac procedures were required. Antegrade pulmonary blood flow was closed in most patients. TCPC was performed under cardiopulmonary bypass using an extracardiac nonringed polytetrafluoroethylene graft (Gore-Tex; WL Gore & Associates).^23^ Fenestration was performed only for very high-risk patients, such as those with a one-lung Fontan, severe atrioventricular regurgitation, or impaired systemic ventricular function.

### Statistical Analysis

Categorical variables are presented as absolute numbers and percentages, and continuous variables are expressed as median with interquartile range (IQR). The χ^2^ test was used for categorical data analysis. For continuous variables, normality was assessed using Shapiro-Wilk tests and Q-Q plots. The Student *t* test was used for normally distributed data, and the Mann-Whitney *U* test was used for non-normally distributed data. A paired *t* test was used to compare the PAIs measured at pre-BCPS, pre-TCPC, and post-TCPC. Correlations between continuous variables were assessed using the Pearson correlation coefficient (r). Logistic regression models were used to identify the variables significantly associated with in-hospital morbidities, and odds ratios (ORs) with 95% confidence intervals were estimated. Cox proportional hazard models were used to identify variables significantly associated with mortality and failing Fontan, and hazard ratio (HRs) with 95% confidence intervals were estimated. To construct categorical variables from continuous data, the cutoff point was determined by receiver operating characteristic analysis with the Youden index. All statistical analyses were performed using SPSS version 28.0 for Windows (IBM) and R statistical software (R Foundation for Statistical Computing).

## Results

### Patient Characteristics and PAI

During the study period, 529 patients underwent TCPC at our institution. Of these, 138 were excluded from the present analysis due to unavailable angiography data, inadequate image quality for PA measurement, or missing clinical follow-up. Thus, a total of 391 patients were included in this study. Single right BCPS was performed in 328 patients (83.9%), and 63 patients underwent other types of BCPS (single left BCPS in 16 and bilateral BCPS in 47). Patient characteristics are shown in Table 1. The median age at TCPC was 2.1 years (IQR, 1.7-2.6 years). The most frequent diagnosis was hypoplastic left heart syndrome (HLHS), in 148 patients (37.9%).

**Table 1:** Patient characteristics

Characteristic	Total cases	Right BCPS	Other types	*P* value
Number of patients (%)	391	328 (83.9)	63 (16.1)	
Age at TCPC, y, median (IQR)	2.1 (1.7-2.6)	2.0 (1.7-2.5)	2.4 (1.9-3.0)	.169
Weight at TCPC, kg, median (IQR)	11.4 (10.6-13.0)	11.4 (10.6-13.0)	11.5 (10.5-13.2)	.213
Primary diagnosis, n (%)				
HLHS	148 (37.9)	137 (41.8)	11 (17.5)	**<.001**
UVH	65 (16.6)	33 (10.1)	32 (50.8)	**<.001**
TA	52 (13.3)	46 (14.0)	6 (9.5)	.335
DILV	45 (11.5)	41 (12.5)	4 (6.3)	.161
PAIVS	19 (4.9)	19 (5.8)	0 (0.0)	.050
ccTGA	19 (4.9)	18 (5.5)	1 (1.6)	.187
UAVSD	18 (4.6)	12 (3.7)	6 (9.5)	**.042**
Others	26 (6.6)	22 (6.7)	4 (6.3)	.91
Dominant right ventricle	241 (61.6)	195 (59.5)	46 (73.0)	**.043**
Associated cardiac anomaly, n (%)				
TGA	81 (20.7)	63 (19.2)	18 (28.6)	.093
DORV	47 (12.0)	27 (8.2)	20 (31.7)	**<.001**
CoA	43 (11.0)	36 (11.0)	7 (11.1)	.975
Dextrocardia/situs inversus	34 (8.7)	14 (4.3)	20 (31.7)	**<.001**
Heterotaxy	33 (8.4)	6 (1.8)	27 (42.9)	**<.001**
TAPVC/PAPVC	28 (7.2)	12 (3.7)	16 (25.4)	**<.001**
Systemic venous return anomaly	43 (11.0)	14 (4.3)	29 (46.0)	**<.001**
Stage I palliation, n (%)				
Norwood/DKS	208 (53.2)	189 (57.6)	19 (30.2)	**<.001**
AP shunt	85 (21.7)	71 (21.6)	14 (22.2)	.919
PAB	35 (9.0)	24 (7.3)	11 (17.5)	**.010**
PDA stent	38 (9.7)	31 (9.5)	7 (11.1)	.684
Stage II palliation, n (%)	391 (100)	328 (00)	63 (100)	
Age at BCPS, months, median (IQR)	4.3 (3.3-6.4)	4.1 (3.2-5.8)	5.8 (3.5-12.2)	.235
Weight at BCPS, kg, median (IQR)	5.3 (4.7-6.2)	5.3 (4.6-6.1)	5.7 (4.9-7.0)	.386
APBF open, n (%)	13 (3.3)	13 (4.0)	0 (0.0)	.108

Bold indicates *P* < .05.

*BCPS*, Bidirectional cavopulmonary shunt; *TCPC*, total cavopulmonary connection; *IQR*, interquartile range; *HLHS*, hypoplastic left heart syndrome; *UVH*, univentricular heart; *TA*, tricuspid atresia; *DILV*, double-inlet left ventricle; *ccTGA*, congenitally corrected transposition of the great arteries; *PAIVS*, pulmonary atresia and intact ventricular septum; *UAVSD*, unbalanced atrioventricular septal defect; *TGA*, transposition of the great arteries; *DORV*, double-outlet right ventricle; *CoA*, coarctation of the aorta; *TAPVC*, total anomalous pulmonary venous connection; *PAPVC*, partial anomalous pulmonary venous connection; *AP*, aortopulmonary; *PAB*, pulmonary artery banding; *PDA*, patent ductus arteriosus; *APBF*, antegrade pulmonary blood flow.

All patients underwent BCPS, at a median age of 4.3 months (IQR, 3.3-6.4 months). Antegrade pulmonary blood flow was left open at BCPS in 13 patients (3.3%), and a left PA stent was implanted in 52 patients (13.3%) before TCPC. Pre-BCPS and pre-TCPC cardiac catheterization data are shown in Table E1. Pre-TCPC, the left PAI (median, 86 mm^2^/m^2^ vs 57 mm^2^/m^2^; *P* < .001), left-to-right ratio (0.95 vs 0.57; *P* < .001), and PA symmetry index (0.67 vs 0.57; *P* = .026) were lower in patients with right-sided BCPS compared to those with other types of BCPS.

### Perioperative and Follow-up Data

The median lengths of stay in the intensive care unit (7 days vs 5 days; *P* = .051) and in the hospital (21 days vs 18 days, *P* = .065) were similar between patients with right-sided BCPS and those with other types of BCPS (Table E2). There were 6 in-hospital mortalities. Ascites requiring drainage occurred more frequently in patients with other types of BCPS (27.0% vs 15.0%; *P* = .021), whereas the prevalences of prolonged (>7 days) pleural effusion (57.1% vs 57.4%; *P* = .974), chylothorax (30.2% vs 24.2%; *P* = .321), and secondary fenestration (defined by late creation of fenestration after TCPC) (1.6% vs 0.9%; *P* = .627) were similar in the 2 groups. PAI and PA symmetry index were similar in patients with antegrade pulmonary blood flow (Table E3) and between patients with and patients without fenestration (Table E4). The median duration of follow-up was 7.5 years (IQR, 4.3-12.7 years). Transplant-free survival was similar in patients with right BCPS and those with other types of BCPS (97.4% vs 93.3% at 10 years; *P* = .181).

### Changes in PAI and PA Symmetry Index

**Figure 1:**
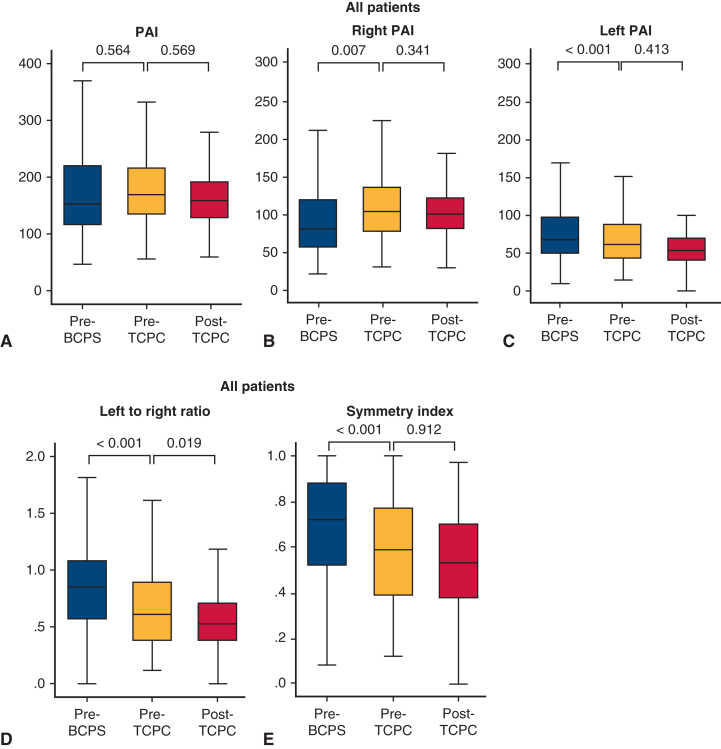
Changes in the pulmonary artery index (*PAI*) during and after staged Fontan completion in all patients. In the box-and-whisker graphs, the *upper* and *lower borders* of the box indicate the upper and lower quartiles, the *middle horizontal line* represents the median, and the upper and lower whiskers represent the maximum and minimum values, respectively.

**Figure 2:**
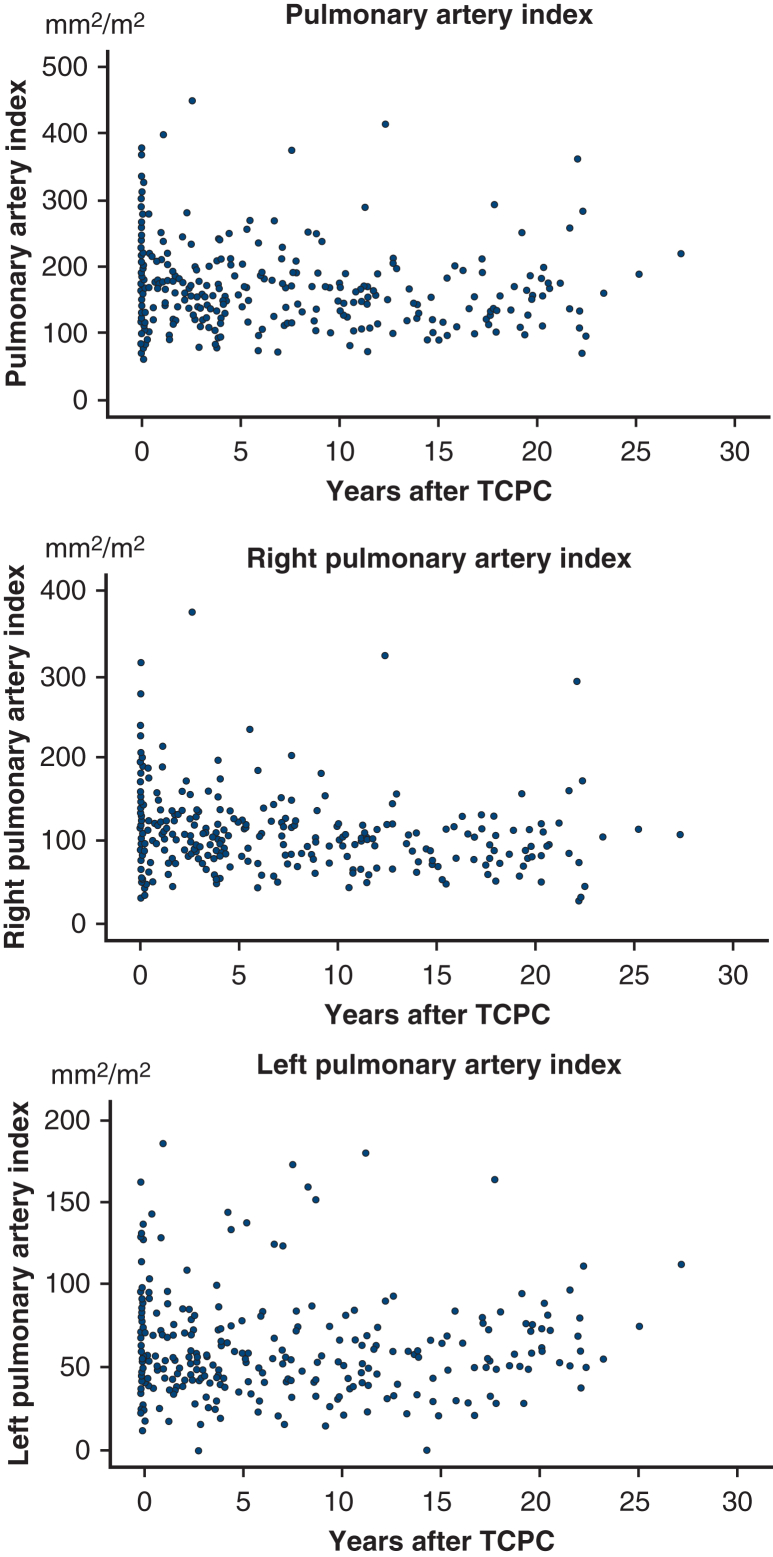
Scatter diagrams displaying the relationship of time after total cavopulmonary connection (*TCPC*) and the pulmonary artery index (*PAI*) and pulmonary artery (*PA*) symmetry indices.

PAI and PA symmetry index values at BCPS, at TCPC, and after TCPC are summarized in Table 2 and Figures E1-E3. Serial changes for all patients are shown in Figure 1. The PAI did not change between BCPS and TCPC (*P* = .564) or between pre- and post-TCPC (*P* = .569). The right PAI increased significantly between BCPS and TCPC (*P* = .007), while the left PAI decreased (*P* < .001). Both remained stable between pre-TCPC and post-TCPC (right, *P* = .341; left, *P* = .413). The left-to-right ratio decreased from pre-BCPS to pre-TCPC (*P* < .001) and from pre-TCPC to post-TCPC (*P* = .019). The PA symmetry index also decreased significantly between pre-BCPS and pre-TCPC (*P* < .001) but did not change thereafter (*P* = .912). These changes were more pronounced in patients with right BCPS (Figure E4). Serial changes in patients with right, left, and bilateral BCPS are shown in Figure E5 and Table E5. The 3 groups demonstrated distinct profiles regarding changes in PAIs and PA symmetry indices. Postoperative yearly distributions of PAIs are shown in Figure 2. Right PAI correlated negatively with follow-up duration (r = −0.146; *P* = .007), whereas PAI (r = −0.096; *P* = .077) and left PAI (r = 0.028; *P* = .604) did not.

**Table 2:** Longitudinal changes in PAI and PA balance

Variables	Right BCPS	Other types of BCPS			*P* value, left and bilateral
		All other types	Single left BCPS	Bilateral BCPS	
No. of patients	328 (83.9)	63 (16.1)	16	47	
Pre-BCPS, median (IQR)					
PAI	149 (110-207)	176 (143-309)	159 (146-322)	215 (133-316)	.749
Right PAI	78 (57-115)	92 (70-176)	78 (69-136)	96 (72-179)	.358
Left PAI	65 (47-89)	90 (58-140)	61 (63-181)	88 (56-140)	.565
Left-to-right ratio	0.83 (0.56-1.07)	0.98 (0.74-1.22)	1.03 (0.88-1.58)	0.90 (0.67-1.15)	.209
Symmetry index	0.71 (0.52-0.88)	0.78 (0.53-0.90)	0.79 (0.56-0.94)	0.77 (0.52-0.90)	.733
Pre-TCPC, median (IQR)					
PAI	165 (131-208)	188 (142-240)	158 (110-225)	189 (155-250)	.183
Right PAI	105 (81-138)	97 (61-128)	77 (51-130)	103 (74-128)	.181
Left PAI	59 (42-82)	85 (60-115)	76 (58-115)	86 (61-115)	.734
Left-to-right ratio	0.57 (0.39-0.81)	0.95 (0.55-1.38)	0.96 (0.69-1.49)	0.92 (0.49-1.38)	.244
Symmetry index	0.57 (0.39-0.77)	0.67 (0.48-0.84)	0.69 (0.47-0.91)	0.64 (0.48-0.80)	.442
Post-TCPC, median (IQR)					
PAI	157 (129-181)	163 (139-229)	185 (127-266)	163 (141-223)	.507
Right PAI	103 (82-123)	100 (68-114)	80 (51-117)	101 (87-127)	.282
Left PAI	52 (41-63)	75 (49-89)	86 (70-166)	67 (41-86)	**.036**
Left-to-right ratio	0.49 (0.37-0.68)	0.70 (0.55-1.10)	1.50 (0.86-1.68)	0.63 (0.53-0.87)	**<.001**
Symmetry index	0.49 (0.37-0.68)	0.62 (0.55-0.83)	0.61 (0.58-0.81)	0.63 (0.53-0.84)	.668

Bold indicates *P* < .05.

PAI is expressed in mm^2^/m^2^. *PAI*, Pulmonary artery index; *PA*, pulmonary artery; *BCPS*, bidirectional cavopulmonary shunt; *IQR*, interquartile range; *TCPC*, total cavopulmonary connection.

### Impact of PA Size on Postoperative Morbidity, Survival, and Failing Fontan

**Figure 3:**
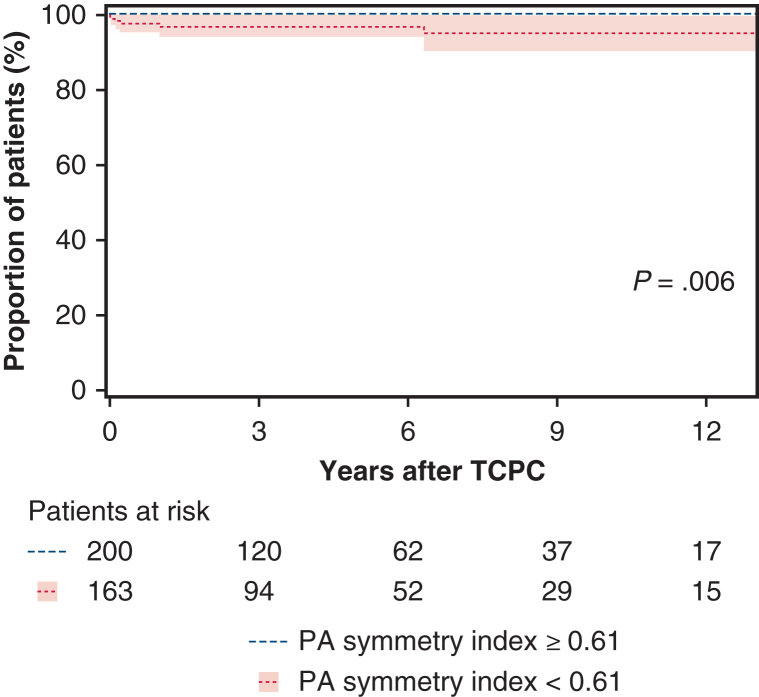
Transplant-free survival after total cavopulmonary connection (*TCPC*) in terms of the pulmonary artery (*PA*) symmetry index. Shaded areas indicate 95% confidence interval.

A summary of risk factor analysis with pre-TCPC variables is shown in Table 3. A pre-TCPC lower PAI (*P* = .009; OR, 0.995) was a risk for prolonged pleural effusion. No pre-TCPC variables were identified as a risk for chylothorax. A pre-TCPC lower PAI (*P* = .005; OR, 0.990) and pre-TCPC higher PA pressure (PAP) (*P* = .008; OR, 1.172) were risk factors for ascites. A pre-TCPC low symmetry index was a risk factor for mortality (*P* = .032; HR, 12.930). Receiver operating characteristic analysis identified a PA symmetry index cutoff value of 0.61 (sensitivity, 83%; specificity, 57%; area under the curve, 0.753), which significantly discriminated transplant-free survival (*P* = .006; Figure 3). No PAI-related variable was associated with failing Fontan; however, pre-TCPC higher PAP was a risk factor (*P* = .020; HR, 1.202). Results of risk factor analysis with all variables are provided in Tables E6-E10.

**Table 3:** Impact of PAI and other variables on outcomes after TCPC (all patients)

Variable	Univariate			Multivariate		
	*P* value	OR/HR	95% CI	*P* value	OR/HR	95% CI
Pleural effusion		OR			OR	
PAI	**.006**	0.996	0.992-0.999	**.009**	0.995	0.992-0.999
Right PAI	.159	0.997	0.992-1.001			
Left PAI	**.003**	0.991	0.985-0.997			
Symmetry index	.085	0.458	0.188-1.114			
PAP	.254	1.051	0.965-1.145			
TPG	.652	1.031	0.904-1.175			
Ventricular dysfunction	.864	1.057	0.563-1.981			
Significant AVVR	**.038**	1.223	1.011-1.480			
Ascites		OR			OR	
PAI	**.015**	0.994	0.989-0.999	**.005**	0.990	0.982-0.997
Right PAI	**.004**	0.989	0.982-0.996			
Left PAI	.420	0.997	0.989-1.005			
Symmetry index	.934	0.953	0.303-3.002			
PAP	**.007**	1.162	1.042-1.296	**.008**	1.172	1.043-1.318
TPG	.085	1.156	0.980-1.364			
Ventricular dysfunction	.440	1.315	0.656-2.633			
Significant AVVR	.098	1.223	0.963-1.551			
Survival		HR			HR	
PAI	.522	0.995	0.981-1.010			
Right PAI	.129	0.981	0.956-1.006			
Left PAI	.483	1.007	0.988-1.026			
Symmetry index	**.032**	12.930	1.238-133.023	**.032**	12.930	1.238-133.023
PAP	.873	1.028	0.735-1.438			
TPG	.276	1.275	0.824-1.973			
Ventricular dysfunction	.799	0.169	0.000-41553.3			
Significant AVVR	.818	0.918	0.443-1.902			
Failing Fontan		HR			HR	
PAI	.311	0.997	0.991-1.003			
Right PAI	.874	0.999	0.991-1.007			
Left PAI	.186	0.992	0.981-1.004			
Symmetry index	.550	0.631	0.140-2.853			
PAP	**<.001**	1.229	1.090-1.385	**.020**	1.202	1.030-1.403
TPG	**.003**	1.351	1.111-1.642			
Ventricular dysfunction	.404	0.103	0.000-21.571			
Significant AVVR	.807	0.961	0.701-1.319			

Bold indicates *P* < .05.

*PAI*, Pulmonary artery index; *TCPC*, total cavopulmonary connection; *OR*, odds ratio; *HR*, hazard ratio; *CI*, confidence interval; *PAP*, pulmonary artery pressure; *TPG*, transpulmonary gradient; *AVVR*, atrioventricular valve regurgitation.

### Impact of PA Size on Exercise Capacity

In the analysis of all patients, pre-TCPC PAP was negatively correlated with peak oxygen consumption (VO_2_) (r = −0.471; *P* = .011), but no PAI-related variable had a significant correlation (Table E11). In patients with right-sided BCPS, post-TCPC PA symmetry index (r = −0.599, *P* = .040) and left-to-right ratio (r = −0.599, *P* = .040) were negatively correlated with peak VO_2_.

## Discussion

### Summary of Results

**Figure 4:**
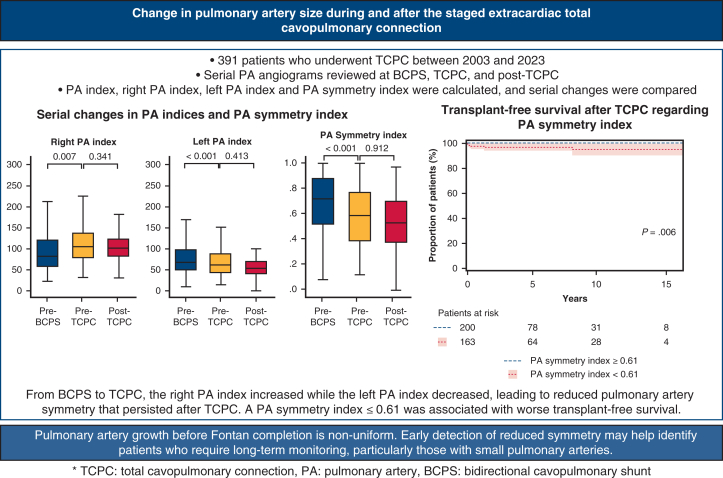
Graphical abstract summarizing the study design, key findings, and clinical implications.

The PAI did not change during or after the staged TCPC completion; however, the right PAI increased between BCPS and TCPC, whereas the left PAI decreased. Pre-TCPC low PA symmetry index was a risk factor for mortality, with a cutoff value of 0.61 discriminating transplant-free survival, and pre-TCPC low PAI was a risk factor for postoperative prolonged pleural effusion and ascites (Figure 4, Video 1).

### PA Development During Staged Fontan Completion

PA development before the Fontan procedure has been widely discussed. Historically, well-developed PA size and absence of distortion are key requirements for the Fontan procedure and are included in the “ten commandments” of the original Fontan criteria.^24^ Since the introduction of the staged Fontan strategy, PA size has not been a significant focus at Fontan completion. In patients who survived BCPS and reached assessment for Fontan completion, PAP was typically ≤10 mm Hg, and PA size was acceptable in most. After stage II, pulmonary blood flow becomes passive, but continued PA development remains necessary. Previous studies reported no significant change in PAI between BCPS and TCPC.^9^^,^^10^ In the present study, we observed an increase in the right PAI and a decrease in the left PAI after BCPS, along with a decrease in the left-to-right PAI ratio between BCPS and TCPC. These changes were more pronounced in patients with right-sided BCPS, which may reflect preferential right SVC flow and left PA compression by adjacent structures. The adverse impact of a small left PA on outcomes has been reported in previous studies^7^^,^^25^; therefore, achieving balanced PA development between BCPS and TCPC appears to be an important issue.

### Changes in PA Size After Fontan Completion

Changes in PA size after the Fontan procedure remain controversial. Several studies have demonstrated that post-TCPC PA growth is disproportionate to somatic growth,^11^^,^^12^^,^^17^^,^^26^ whereas others have found it to be proportional to the change in body surface area.^18^ Ghosh and colleagues^17^ reported a stable indexed right PA area but a decreased indexed left PA area. Similarly, Restrepo and colleagues^26^ observed stable right PA size and decreased left PA size over time after the Fontan operation. Our findings closely resemble those results. We speculate that 2 factors contribute to this result. First, the right-sided anastomosis of the extracardiac conduit may direct inferior vena cava flow preferentially to the right lung. Although we place the conduit as far left as possible, this is limited by cardiac motion and adhesions between the left PA and the aorta. Second, a reconstructed aorta has external anatomic limitations on left PA growth.^27^ In this study, more than one-half of the patients underwent the Norwood procedure as stage I palliation, which may prevent recovery of early vessel growth deficits. Thus, impaired left PA growth is likely due to both physiologic and morphologic factors, and the left PA may be a target for improved surgical planning.

### Impact of PA Size and PA Symmetry on Outcomes Following TCPC

Several studies have investigated the impact of a small left PA on outcomes after the Fontan procedure. Kido and colleagues^8^ reported that left PA stenosis following right-sided BCPS was associated with chylothorax and longer intensive care unit stay after TCPC. Mercer-Rosa and colleagues^25^ demonstrated that smaller left PA diameter at Fontan was associated with worse quality of life, and Alsaied and colleagues^28^ linked maldistribution of PA flow from left PA stenosis to reduced exercise capacity in adult patients with Fontan circulation. Consistently, in our study patients, pre-TCPC small left PA was associated with prolonged pleural effusion following TCPC. Regarding late outcomes, a lower PA symmetry index, with a cutoff of 0.61, was a risk factor for mortality. Notably, in patients with right-sided BCPS who underwent exercise testing, post-TCPC PA symmetry index was negatively correlated with peak VO_2_, a counterintuitive finding warranting further investigation. Nevertheless, achieving a PA symmetry index >0.61 may represent a new “commandment” for Fontan completion. While these results do not establish a strong causal relationship between a small left PA and late morbidity, our findings suggest a clinically relevant association between a small left PA and adverse outcomes. The details and impact of surgical/catheter interventions on the left PA on outcomes merit further investigation.^29^

### Impact of SVC Anatomy

In this study, analysis of serial changes in PA size in relation to SVC anatomy revealed quite different profiles of PA development. Ipsilateral PA development was dominant on the SVC side. Most patients had a right-sided SVC, but some had a bilateral or left-sided SVC, leading to bilateral or left-sided BCPS. PA development should be evaluated differently in patients with these variations compared to patients with a right-sided SVC. It is important to further elucidate the impact of PA growth pattern, including its effects on the hemodynamic efficiency of the TCPC, and to understand how PA growth heterogeneity may influence overall hemodynamic performance. However, the main issue, particularly in HLHS patients, is left PA growth.

### Limitations

This retrospective, single-center study has some inherent limitations. The large number of excluded patients may have introduced selection bias. Patients with poor or excellent clinical status might not have undergone follow-up cardiac catheterization, leading to missing data likely related to outcomes. Finally, this study spans a long period, during which evolving treatment strategies, including anticoagulation protocols and PA stenting techniques, may have influenced long-term outcomes.

## Conclusions

In our cohort, the right PAI increased between BCPS and TCPC, whereas the left PAI and PA symmetry index decreased, indicating progressive imbalance of pulmonary blood flow favoring the right lung. Following TCPC, the PA indices remained stable. Growth patterns differed by SVC anatomy, with the PA ipsilateral to the SVC growing more than the contralateral side. Notably, a PA symmetry index <0.61 was identified as a risk factor for mortality following TCPC. These findings highlight the importance of achieving balanced PA growth prior to Fontan completion and the need for long-term monitoring and potential interventions in patients with asymmetric PA development.

## Conflict of Interest Statement

The authors reported no conflicts of interest.

The *Journal* policy requires editors and reviewers to disclose conflicts of interest and to decline handling or reviewing manuscripts for which they may have a conflict of interest. The editors and reviewers of this article have no conflicts of interest.

## Figures and Tables

**FigureE1A:**
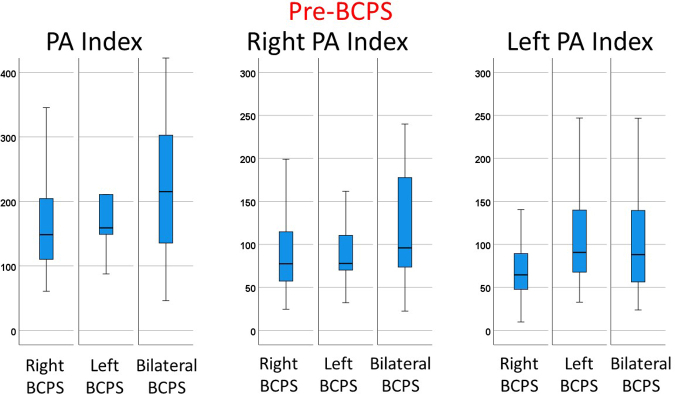


**FigureE1B:**
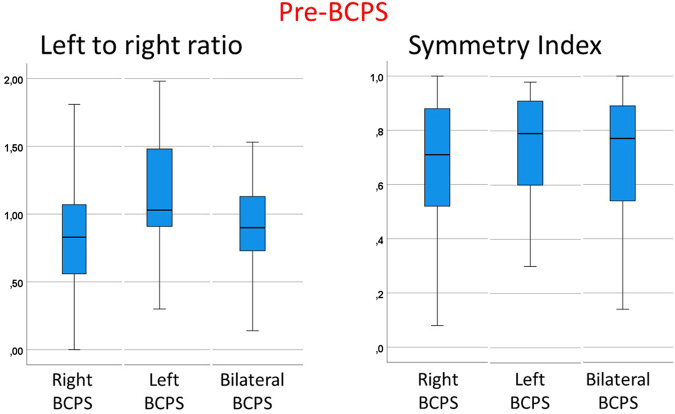


**FigureE2A:**
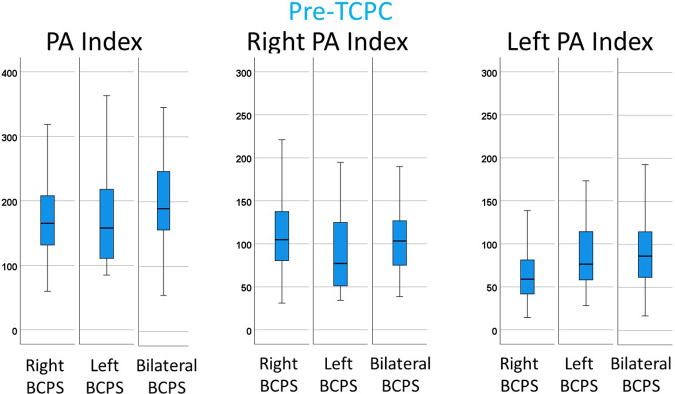


**FigureE2B:**
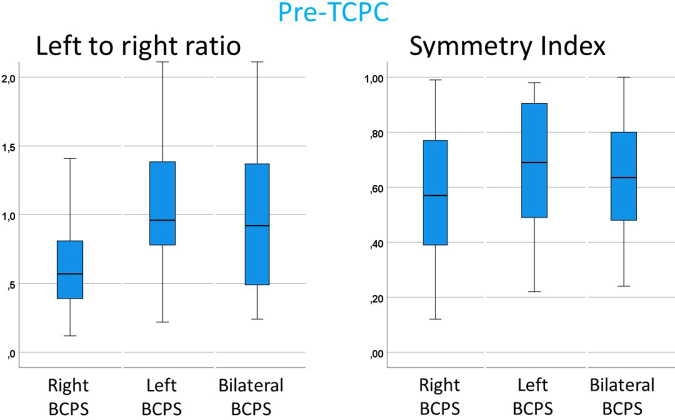


**FigureE3A:**
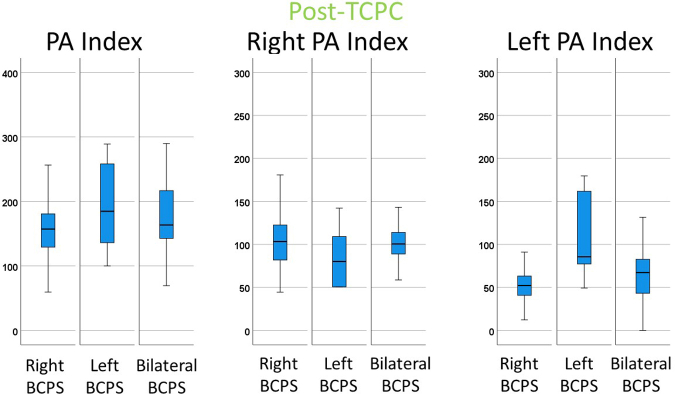


**FigureE3B:**
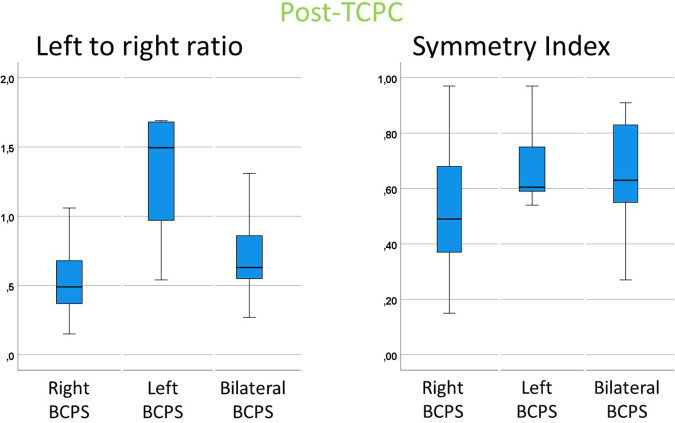


**FigureE4ABC:**
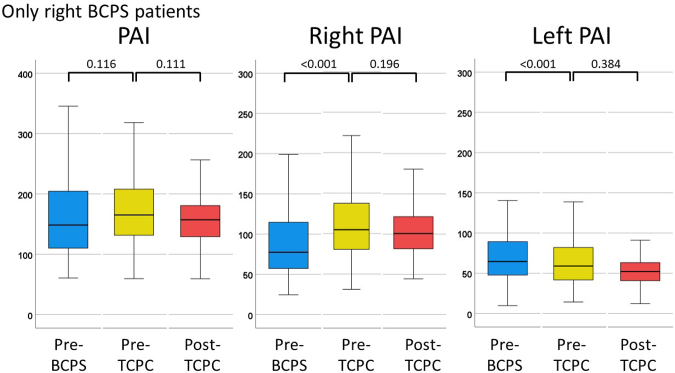


**FigureE4DE:**
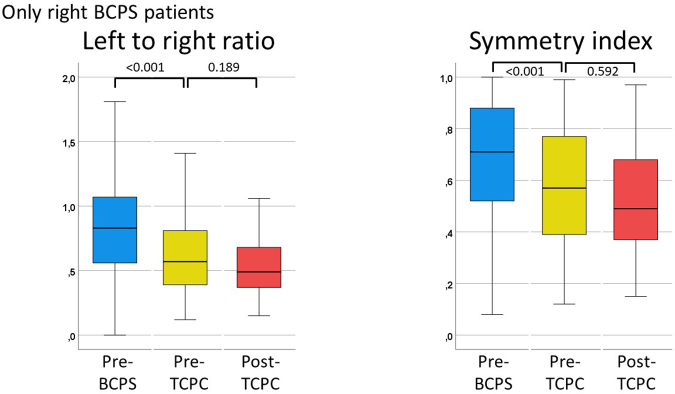


**FigureE5A1PAI:**
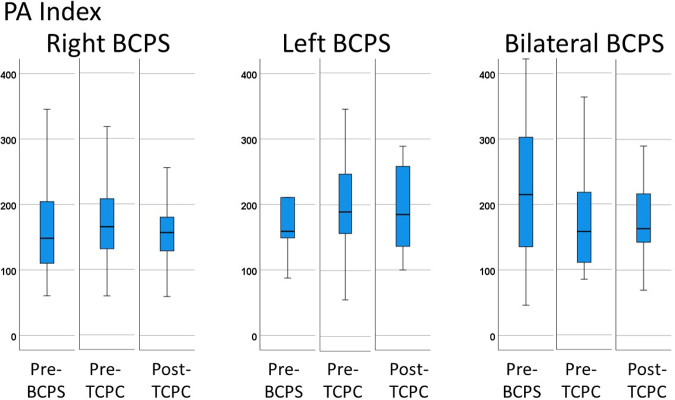


**FigureE5B2RPAI:**
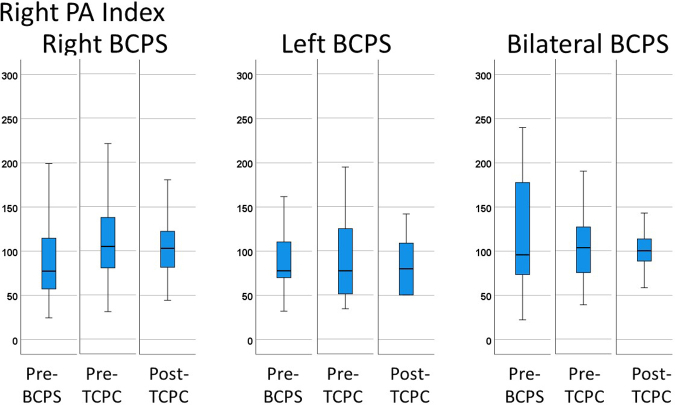


**FigureE5C3LPAI:**
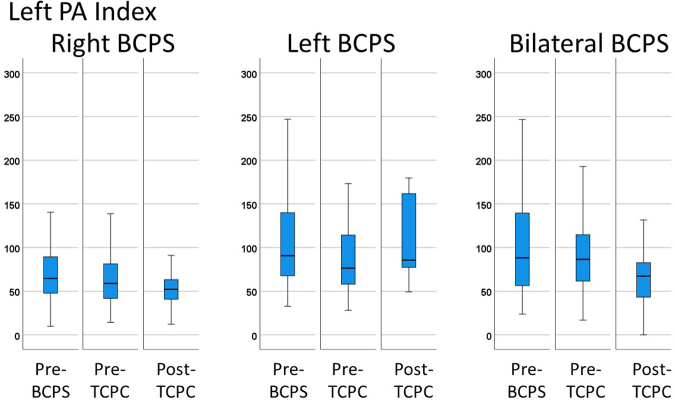


**FigureE5D4LtoR:**
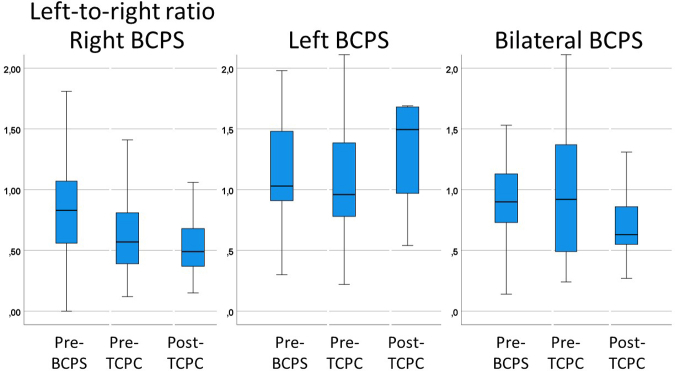


**FigureE5E5Sym:**
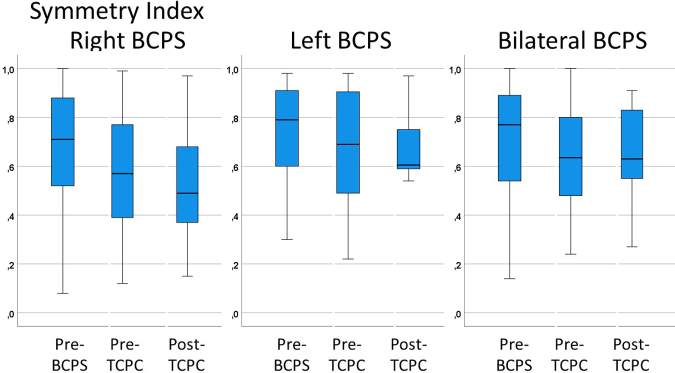


## Supplementary Data

Online Data Supplement 1

Video 1 The lead author briefly explains the study's importance and relevance. Video available at: https://www.jtcvs.org/article/S2666-2736(26)00144-0/fulltext.
