# Supplementary material for: Changes in pulmonary artery size during and after staged extracardiac total cavopulmonary connection
Source: JTCVS Open. 2026 Mar 12;31:101721. doi: 10.1016/j.xjon.2026.101721 (PMC13316346; doi:10.1016/j.xjon.2026.101721)
Supplement: Online Data Supplement 1 [file mmc1.docx]

**Supplementary Tables**

**Supplementary Table E1**

| Table E1. Cardiac catheterization data before BCPS and before TCPC | | | | |  |
| --- | --- | --- | --- | --- | --- |
| Variables: | | Total cases | Right BCPS | Other types | p-value |
| Number of patients | | 391 | 328 (83.9) | 63 (16.1) |  |
| **Before BCPS** | |  |  |  |  |
|  | Hemoglobin (g/dl) | 13.9 (12.4-16.3) | 13.9 (12.4-15.3) | 15.5 (141.-16.9) | 0.597 |
| Hemodynamic | |  |  |  |  |
|  | PAP (mmHg) | 14 (11-17) | 14 (12-17) | 10 (8-12) | 0.964 |
|  | LAP (mmHg) | 6 (5-8) | 6 (5-8) | 6 (4-8) | 0.601 |
|  | TPG (mmHg) | 7 (5-10) | 7 (5-10) | 4 (3-5) | 0.365 |
|  | SVP (mmHg) | 79 (72-86) | 79 (72-86) | 86 (79-96) | 0.519 |
|  | EDP (mmHg) | 9 (7-11) | 9 (7-11) | 8 (6-10) | 0.400 |
|  | MAP (mmHg) | 49 (45-54) | 49 (45-54) | 59 (52-65) | 0.166 |
|  | SO2 (%) | 76 (72-80) | 76 (71-80) | 82 (80-86) | 0.259 |
| PA size & balance | |  |  |  |  |
|  | PAI (mm^2^/m^2^) | 153 (116-222) | 149 (110-207) | 178 (143-309) | **<0.001** |
|  | Right PAI (mm^2^/m^2^) | 81 (58-121) | 78 (57-115) | 92 (70-176) | **0.016** |
|  | Left PAI (mm^2^/m^2^) | 67 (49-97) | 65 (47-89) | 90 (58-140) | **<0.001** |
|  | Left-to-right ratio | 0.85 (0.57-1.08) | 0.83 (0.56-1.07) | 0.98 (0.74-1.22) | **0.038** |
|  | Symmetry index | 0.72 (0.52-0.88) | 0.71 (0.52-0.88) | 0.78 (0.53-0.90) | 0.623 |
| **Before TCPC** | |  |  |  |  |
|  | Hemoglobin (g/dl) | 15.6 (14.4-16.7) | 15.6 (14.3-16.6) | 15.5 (141.-16.9) | 0.214 |
| Hemodynamic | |  |  |  |  |
|  | PAP (mmHg) | 10 (8-11) | 10 (8-11) | 10 (8-12) | 0.564 |
|  | LAP (mmHg) | 6 (4-7) | 6 (4-7) | 6 (4-8) | 0.286 |
|  | TPG (mmHg) | 4 (3-5) | 4 (3-5) | 4 (3-5) | 0.115 |
|  | SVP (mmHg) | 85 (77-93) | 84 (77-93) | 86 (79-96) | 0.824 |
|  | EDP (mmHg) | 8 (6-10) | 8 (6-9) | 8 (6-10) | 0.427 |
|  | MAP (mmHg) | 58 (52-64) | 58 (51-63) | 59 (52-65) | 0.150 |
|  | SO2 (%) | 83 (80-86) | 83 (80-86) | 82 (80-86) | 0.199 |
| PA size & balance | |  |  |  |  |
|  | PAI (mm^2^/m^2^) | 169 (133-216) | 165 (131-208) | 188 (142-240) | 0.080 |
|  | Right PAI (mm^2^/m^2^) | 104 (78-136) | 105 (81-138) | 97 (61-128) | 0.217 |
|  | Left PAI (mm^2^/m^2^) | 61 (44-88) | 59 (42-82) | 86 (60-115) | **<0.001** |
|  | Left-to-right ratio | 0.61 (0.39-0.89) | 0.57 (0.39-0.81) | 0.95 (0.55-1.38) | **<0.001** |
|  | Symmetry index | 0.59 (0.39-0.77) | 0.57 (0.39-0.77) | 0.67 (0.48-0-84) | **0.026** |
| Data are shown by N (%) or median (IQR) | | |  |  |  |

PAP: pulmonary artery pressure, LAP: left atrial pressure, TPG: trans-pulmonary gradient, SVP: systolic ventricular pressure, EDP: end-diastolic pressure, MAP: mean arterial pressure, PAI: pulmonary artery index

**Supplementary Table E2**

| Table E2. Perioperative variables | | |  |  |  |
| --- | --- | --- | --- | --- | --- |
| Variables | | Total cases | Right BCPS | Other types | p-value |
| Number of patients | | 391 | 328 (83.9) | 63 (16.1) |  |
| **Operative data** | |  |  |  |  |
| Conduit diameter (mm) | |  |  |  |  |
|  | 14 | 1 (0.3) | 0 (0.0) | 1 (1.6) | 0.116 |
|  | 16 | 4 (1.0) | 2 (0.6) | 2 (3.2) |  |
|  | 18 | 368 (94.1) | 311 (94.8) | 57 (90.5) |  |
|  | 20 | 18 (4.6) | 15 (4.6) | 3 (4.8) |  |
| CPB time (minutes) | | 64 (47-89) | 62 (46-85) | 77 (57-104) | **0.002** |
| Cardiac arrest | | 70 (17.9) | 49 (14.9) | 21 (33.3) | **<0.001** |
| Arrest time (minutes) | | 36 (22-55) | 35 (24-61) | 37 (20-50) | 0.225 |
| Fenestration at TCPC | | 29 (7.4) | 25 (7.6) | 4 (6.3) | 0.724 |
| Concomitant procedure | | 75 (19.2) | 58 (17.7) | 17 (27.0) | 0.086 |
|  | DKS | 8 (2.0) | 8 (2.4) | 0 (0.0) | 0.210 |
|  | AVV procedure | 42 (10.7) | 31 (9.5) | 11 (17.5) | 0.060 |
|  | PA reconstruction | 13 (3.3) | 10 (3.0) | 3 (4.8) | 0.487 |
|  | SAS/VSD enlargement | 8 (2.0) | 5 (1.5) | 3 (4.8) | 0.096 |
|  | Pacemaker implant | 8 (2.0) | 7 (2.1) | 1 (1.6) | 0.779 |
| **Postoperative data** | |  |  |  |  |
| ICU stay (days) | | 6 (4-8) | 5 (4-8) | 7 (4-11) | 0.051 |
| Hospital stay (days) | | 19 (13-26) | 18 (13-26) | 21 (14-29) | 0.065 |
| Complications | |  |  |  |  |
|  | Pleural effusion | 223 (57.3) | 187 (57.4) | 36 (57.1) | 0.974 |
|  | Chylothorax | 98 (25.2) | 79 (24.2) | 19 (30.2) | 0.321 |
|  | Ascites | 66 (7.0) | 49 (15.0) | 17 (27.0) | **0.021** |
| Secondary fenstration | | 4 (1.0) | 3 (0.9) | 1 (1.6) | 0.627 |
| Variables were presented in N (%) or median (IQR) | | | |  |  |
| PA: pulmonary artery; TCPC: total cavopulmonary connection | | | |  |  |
| CPB: cardiopulmonary bypass; | | | |  |  |
| DKS: Dames-Kaye-Stansel anastomosis; AVV: atrioventricular valve | | | | |  |
| PA: pulmonary artery; SAS: subaortic stenosis; VSD: ventricular septal defect | | | | |  |
| ICU: intensive care unit | |  |  |  |  |

**Supplementary Table E3**

| Table E3. Comparison of PA index between patients with and without APBF | | | | |
| --- | --- | --- | --- | --- |
| Variables | | APBF (-) | APBF (+) | p-value |
| **All patients** | |  |  |  |
|  | Number of patients | 378 (96.7) | 13 (3.3) |  |
|  | PAI | 168 (132-215) | 173 (152-292) | 0.056 |
|  | Right PAI | 104 (77-136) | 113 (94-201) | 0.083 |
|  | Left PAI | 61 (43-87) | 83 (49-94) | 0.225 |
|  | Left-to-right ratio | 0.61 (0.39-0.90) | 0.60 (0.41-0.86) | 0.519 |
|  | Symmetry index | 0.58 (0.39-0.77) | 0.60 (0.41-0.86) | 0.608 |
| **Right BCPS** | |  |  |  |
|  | Number of patients | 315 | 13 |  |
|  | PAI | 163 (131-208) | 173 (152-292) | **0.036** |
|  | Right PAI | 105 (79-137) | 113 (94-201) | 0.100 |
|  | Left PAI | 59 (41-80) | 83 (49-94) | 0.073 |
|  | Left-to-right ratio | 0.57 (0.38-0.81) | 0.60 (0.41-0.86) | 0.914 |
|  | Symmetry index | 0.57 (0.38-0.77) | 0.60 (0.41-0.86) | 0.482 |
| **Other types of BCPS** | |  |  |  |
|  | Number of patients | 63 | 0 |  |
|  | PAI | 188 (142-240) |  |  |
|  | Right PAI | 97 (61-128) |  |  |
|  | Left PAI | 86 (60-115) |  |  |
|  | Left-to-right ratio | 0.95 (0.55-1.38) |  |  |
|  | Symmetry index | 0.67 (0.48-0.84) |  |  |
| PAI is expressed by mm^2^/m^2^ | | |  |  |

**Supplementary Table E4**

| Table E4. Comparison of PA index between patients with and without fenestration | | | | |
| --- | --- | --- | --- | --- |
| Variables | | Fenestration (-) | Fenestration (+) | p-value |
| **All patients** | |  |  |  |
|  | Number of patients | 362 (92.6) | 29 (7.4) |  |
|  | PAI | 168 (135-216) | 182 (127-197) | 0.805 |
|  | Right PAI | 104 (78-137) | 112 (75-130) | 0.956 |
|  | Left PAI | 61 (43-88) | 65 (46-94) | 0.651 |
|  | Left-to-right ratio | 0.61 (0.39-0.90) | 0.60 (0.49-0.78) | 0.629 |
|  | Symmetry index | 0.58 (0.39-0.77) | 0.60 (0.49-0.77) | 0.976 |
| **Right BCPS** | |  |  |  |
|  | Number of patients | 303 | 25 |  |
|  | PAI | 164 (132-213) | 173 (126-195) | 0.493 |
|  | Right PAI | 105 (81-138) | 104 (75-128) | 0.279 |
|  | Left PAI | 59 (42-81) | 65 (39-94) | 0.898 |
|  | Left-to-right ratio | 0.57 (0.38-0.81) | 0.65 (0.50-0.84) | 0.420 |
|  | Symmetry index | 0.56 (0.38-0.77) | 0.61 (0.50-0.77) | 0.546 |
| **Other types of BCPS** | |  |  |  |
|  | Number of patients | 59 | 4 |  |
|  | PAI | 187 (142-234) | 224 (143-320) | 0.320 |
|  | Right PAI | 96 (60-126) | 146 (88-246) | **0.020** |
|  | Left PAI | 86 (60-115) | 64 (55-88) | 0.278 |
|  | Left-to-right ratio | 0.96 (0.61-1.40) | 0.54 (0.30-0.66) | 0.083 |
|  | Symmetry index | 0.69 (0.48-0.85) | 0.54 (0.30-0.66) | 0.167 |
| PAI is expressed by mm^2^/m^2^ | | |  |  |

**Supplementary Table E5**

| Table E5. Longitudinal changes in PA index | | | |  |  |  |
| --- | --- | --- | --- | --- | --- | --- |
| Variables | | Pre-BCPS | p-value | Pre-TCPC | p-value | Post-TCPC |
| **All patients** | |  |  |  |  |  |
|  | PAI | 153 (116-222) | 0,564 | 168 (133-216) | 0,569 | 158 (129-191) |
|  | Right PAI | 81 (58-121) | **0,007** | 104 (78-136) | 0,341 | 101 (82-122) |
|  | Left PAI | 67 (49-97) | **0.001** | 61 (44-88) | 0,413 | 53 (41-70) |
|  | Left-to-right ratio | 0.85 (0.57-1.08) | **<0,001** | 0.61 (0.39-0.89) | **0,019** | 0.53 (0.38-0.71) |
|  | Symmetry index | 0.72 (0.52-0.88) | **<0,001** | 0.59 (0.39-0.77) | 0,912 | 0.53 (0.38-0.70) |
| **Right BCPS** | |  |  |  |  |  |
|  | PAI | 149 (110-207) | 0,116 | 165 (131-208) | 0,111 | 157 (129-181) |
|  | Right PAI | 78 (57-115) | **<0,001** | 105 (81-138) | 0,196 | 103 (82-123) |
|  | Left PAI | 65 (47-89) | **<0,001** | 59 (42-82) | 0,384 | 52 (41-63) |
|  | Left-to-right ratio | 0.83 (0.56-1.07) | **<0,001** | 0.57 (0.39-0.81) | 0,189 | 0.49 (0.37-0.68) |
|  | Symmetry index | 0.71 (0.52-0.88) | **<0,001** | 0.57 (0.39-0.77) | 0,582 | 0.49 (0.37-0.68) |
| **Left BCPS** | |  |  |  |  |  |
|  | PAI | 159 (146-322) | 0,082 | 158 (110-225) | 0,088 | 185 (127-266) |
|  | Right PAI | 78 (69-136) | 0,245 | 77 (51-130) | 0,772 | 80 (51-117) |
|  | Left PAI | 91 (63-181) | 0,079 | 76 (58-115) | 0,347 | 86 (70-166) |
|  | Left-to-right ratio | 1.03 (0.88-1.58) | 0,42 | 0.96 (0.69-1.49) | 0,566 | 1.50 (0.86-1.68) |
|  | Symmetry index | 0.79 (0.56-0.94) | 0,553 | 0.69 (0.47-0.91) | 0,209 | 0.61 (0.58-0.81) |
| **Bilateral BCPS** | |  |  |  |  |  |
|  | PAI | 215 (133-316) | **0,027** | 189 (155-250) | **0,022** | 163 (141-223) |
|  | Right PAI | 96 (72-179) | 0,059 | 103 (74-128) | 0,574 | 101 (87-127) |
|  | Left PAI | 88 (56-140) | 0,06 | 86 (61-115) | **0,007** | 67 (41-86) |
|  | Left-to-right ratio | 0.90 (0.67-1.15) | 0,659 | 0.92 (0.49-1.38) | 0,055 | 0.63 (0.53-0.87) |
|  | Symmetry index | 0.77 (0.52-0.90) | 0,28 | 0.64 (0.48-0.80) | 0,461 | 0.63 (0.53-0.84) |
| PAI is expressed in mm^2^/m^2^ | | |  |  |  |  |

**Supplementary Table E6**

Impact of PAI on prolonged pleural effusion after TCPC

| Table E6a. Impact of PAI on prolonged pleural effusion after TCPC (all patients) | | | | | | |
| --- | --- | --- | --- | --- | --- | --- |
| Variables | Univariate | | | Multivariate | | |
|  | p-value | OR | 95% CI | p-value | OR | 95% CI |
| **Pre-BCPS** |  |  |  |  |  |  |
| PAI | **<0.001** | 0.996 | 0.993-0.998 |  |  |  |
| Right PAI | **<0.001** | 0.993 | 0.989-0.997 | **<0.001** | 0.993 | 0.989-0.997 |
| Left PAI | **0.019** | 0.994 | 0.990-0.999 |  |  |  |
| Left-to-right ratio | 0.492 | 1.157 | 0.764-1.751 |  |  |  |
| Symmetry index | **0.031** | 3.006 | 1.107-8.164 |  |  |  |
| Hemoglobin | 0.753 | 0.983 | 0.887-1.091 |  |  |  |
| PAP | 0.247 | 1.035 | 0.977-1.097 |  |  |  |
| TPG | 0.599 | 1.017 | 0.956-1.082 |  |  |  |
| LAP | 0.963 | 1.002 | 0.927-1.083 |  |  |  |
| EDP | 0.489 | 0.974 | 0.905-1.049 |  |  |  |
| SO_2_ | 0.263 | 0.986 | 0.961-1.011 |  |  |  |
| Ventricular dysfunction | 0.151 | 0.634 | 0.226-1.258 |  |  |  |
| Significant AVVR | 0.965 | 1.004 | 0.827-1.220 |  |  |  |
| **Pre-TCPC** |  |  |  |  |  |  |
| PAI | **0.006** | 0.996 | 0.992-0.999 | **0.009** | 0.995 | 0.992-0.999 |
| Right PAI | 0.159 | 0.997 | 0.992-1.001 |  |  |  |
| Left PAI | **0.003** | 0.991 | 0.985-0.997 |  |  |  |
| Left-to-right ratio | 0.290 | 0.775 | 0.483-1.243 |  |  |  |
| Symmetry index | 0.085 | 0.458 | 0.188-1.114 |  |  |  |
| Hemoglobin | 0.079 | 1.103 | 0.989-1.231 |  |  |  |
| PAP | 0.254 | 1.051 | 0.965-1.145 |  |  |  |
| TPG | 0.652 | 1.031 | 0.904-1.175 |  |  |  |
| LAP | 0.673 | 1.020 | 0.929-1.121 |  |  |  |
| EDP | 0.265 | 1.051 | 0.963-1.148 |  |  |  |
| SO_2_ | 0.267 | 0.976 | 0.935-1.019 |  |  |  |
| Ventricular dysfunction | 0.864 | 1.057 | 0.563-1.981 |  |  |  |
| Significant AVVR | **0.038** | 1.223 | 1.011-1.480 |  |  |  |

| Table E6b. Impact of PAI on prolonged pleural effusion after TCPC (right-sided BCPS) | | | | | | |
| --- | --- | --- | --- | --- | --- | --- |
| Variables | Univariate | | | Multivariate | | |
|  | p-value | OR | 95% CI | p-value | OR | 95% CI |
| **Pre-BCPS** |  |  |  |  |  |  |
| PAI | **0.008** | 0.996 | 0.993-0.999 | **0.017** | 0.996 | 0.993-0.999 |
| Right PAI | **0.010** | 0.994 | 0.990-0.999 |  |  |  |
| Left PAI | 0.062 | 0.994 | 0.988-1.000 |  |  |  |
| Left-to-right ratio | 0.753 | 1.080 | 0.668-1.748 |  |  |  |
| Symmetry index | **0.034** | 3.398 | 1.098-10.519 |  |  |  |
| Hemoglobin | 0.582 | 0.968 | 0.864-1.085 |  |  |  |
| PAP | 0.552 | 1.020 | 0.956-1.087 |  |  |  |
| TPG | 0.631 | 1.017 | 0.950-1.089 |  |  |  |
| LAP | 0.759 | 1.014 | 0.926-1.111 |  |  |  |
| EDP | 0.417 | 0.966 | 0.889-1.050 |  |  |  |
| SO_2_ | 0.448 | 0.989 | 0.960-1.018 |  |  |  |
| Ventricular dysfunction | 0.145 | 0.528 | 0.223-1.248 |  |  |  |
| Significant AVVR | 0.948 | 1.007 | 0.819-1.238 |  |  |  |
| **Pre-TCPC** |  |  |  |  |  |  |
| PAI | **0.007** | 0.995 | 0.992-0.999 | **0.008** | 0.995 | 0.990-0.999 |
| Right PAI | 0.133 | 0.996 | 0.991-1.001 |  |  |  |
| Left PAI | **0.002** | 0.989 | 0.982-0.996 |  |  |  |
| Left-to-right ratio | 0.408 | 0.759 | 0.395-1.459 |  |  |  |
| Symmetry index | 0.128 | 0.471 | 0.179-1.242 |  |  |  |
| Hemoglobin | 0.141 | 1.097 | 0.970-1.240 |  |  |  |
| PAP | 0.499 | 1.033 | 0.940-1.136 |  |  |  |
| TPG | 0.781 | 1.020 | 0.885-1.177 |  |  |  |
| LAP | 0.675 | 1.022 | 0.923-1.132 |  |  |  |
| EDP | 0.321 | 1.050 | 0.953-1.157 |  |  |  |
| SO_2_ | 0.507 | 0.984 | 0.940-1.031 |  |  |  |
| Ventricular dysfunction | 0.732 | 1.120 | 0.586-2.140 |  |  |  |
| Significant AVVR | **0.041** | 1.243 | 1.008-1.531 |  |  |  |

| Table E6c. Impact of PAI on prolonged pleural effusion after TCPC (other types of BCPS) | | | | | | |
| --- | --- | --- | --- | --- | --- | --- |
| Variables | Univariate | | | Multivariate | | |
|  | p-value | OR | 95% CI | p-value | OR | 95% CI |
| **Pre-BCPS** |  |  |  |  |  |  |
| PAI | **0.012** | 0.994 | 0.989-0.999 |  |  |  |
| Right PAI | **0.012** | 0.989 | 0.980-0.998 | **0.012** | 0.989 | 0.980-0.998 |
| Left PAI | 0.134 | 0.994 | 0.985-1.002 |  |  |  |
| Left-to-right ratio | 0.391 | 1.461 | 0.614-3.473 |  |  |  |
| Symmetry index | 0.541 | 1.954 | 0.228-16.739 |  |  |  |
| Hemoglobin | 0.647 | 1.061 | 0.625-1.364 |  |  |  |
| PAP | 0.164 | 1.108 | 0.959-1.279 |  |  |  |
| TPG | 0.792 | 1.020 | 0.879-1.184 |  |  |  |
| LAP | 0.713 | 0.972 | 0.838-1.129 |  |  |  |
| EDP | 0.971 | 1.003 | 0.851-1.182 |  |  |  |
| SO_2_ | 0.409 | 0.976 | 0.920-1.035 |  |  |  |
| Ventricular dysfunction | . | . | . |  |  |  |
| Significant AVVR | 0.961 | 0.986 | 0.555-1.752 |  |  |  |
| **Pre-TCPC** |  |  |  |  |  |  |
| PAI | 0.535 | 0.998 | 0.990-1.005 |  |  |  |
| Right PAI | 0.873 | 0.999 | 0.989-1.009 |  |  |  |
| Left PAI | 0.488 | 0.996 | 0984-1.008 |  |  |  |
| Left-to-right ratio | 0.561 | 0.791 | 0.359-1.744 |  |  |  |
| Symmetry index | 0.450 | 0.405 | 0.039-4.212 |  |  |  |
| Hemoglobin | 0.346 | 1.125 | 0.881-1.438 |  |  |  |
| PAP | 0.237 | 1.134 | 0.921-1.397 |  |  |  |
| TPG | 0.629 | 1.086 | 0.777-1.520 |  |  |  |
| LAP | 0.928 | 1.011 | 0.795-1.287 |  |  |  |
| EDP | 0.611 | 1.056 | 0.855-1.305 |  |  |  |
| SO_2_ | 0.217 | 0.930 | 0.829-1.044 |  |  |  |
| Ventricular dysfunction | 1.000 | 1.000 |  |  |  |  |
| Significant AVVR | 0.613 | 1.129 | 0.706-1.804 |  |  |  |

**Supplementary Table E7**

Impact of PAI on chylothorax after TCPC

| Table E7a. Impact of PAI on chylothorax after TCPC (all patients) | | | | |  |  |
| --- | --- | --- | --- | --- | --- | --- |
| Variables |  | Univariate |  |  | Multivariate |  |
|  | p-value | OR | 95% CI | p-value | OR | 95% CI |
| **Pre-BCPS** |  |  |  |  |  |  |
| PAI | **0.023** | 0.997 | 0.994-1.000 |  |  |  |
| Right PAI | **0.005** | 0.993 | 0.988-0.998 | **0.015** | 0.993 | 0.988-0.999 |
| Left PAI | 0.418 | .0998 | 0.992-1.003 |  |  |  |
| Left-to-right ratio | 0.129 | 1.393 | 0.908-2.139 |  |  |  |
| Symmetry index | **0.012** | 4.501 | 1.401-14.454 |  |  |  |
| Hemoglobin | 0.132 | 0.913 | 0.811-1.028 |  |  |  |
| PAP | 0.294 | 1.035 | 0.971-1.104 |  |  |  |
| TPG | 0.962 | 1.002 | 0.933-1.075 |  |  |  |
| LAP | 0.317 | 1.044 | 0.960-1.134 |  |  |  |
| EDP | 0.152 | 1.062 | 0.978-1.154 |  |  |  |
| SO_2_ | 0.666 | 1.006 | 0.979-1.034 |  |  |  |
| Ventricular dysfunction | 0.684 | 1.182 | 0.528-2.646 |  |  |  |
| Significant AVVR | 0.668 | 0.954 | 0.768-1.185 |  |  |  |
| **Pre-TCPC** |  |  |  |  |  |  |
| PAI | 0.727 | 0.999 | 0.996-1.003 |  |  |  |
| Right PAI | 0.819 | 1.001 | 0.996-1.005 |  |  |  |
| Left PAI | 0.386 | 0.997 | 0.990-1.004 |  |  |  |
| Left-to-right ratio | 0.255 | 0.712 | 0.397-1.277 |  |  |  |
| Symmetry index | 0.993 | 1.005 | 0.370-2.724 |  |  |  |
| Hemoglobin | 0.820 | 1.014 | 0.898-1.146 |  |  |  |
| PAP | 0.110 | 1.081 | 0.983-1.189 |  |  |  |
| TPG | 0.882 | 0.989 | 0.849-1.151 |  |  |  |
| LAP | 0.099 | 1.095 | 0.983-1.220 |  |  |  |
| EDP | 0.081 | 1.092 | 0.989-1.205 |  |  |  |
| SO_2_ | 0.413 | 1.021 | 0.972-1.072 |  |  |  |
| Ventricular dysfunction | 0.680 | 1.150 | 0.593-2.230 |  |  |  |
| Significant AVVR | 0.425 | 1.089 | 0.883-1.344 |  |  |  |

| Table E7b. Impact of PAI on chylothorax after TCPC (right-sided BCPS patients) | | | | | | |
| --- | --- | --- | --- | --- | --- | --- |
| Variables |  | Univariate |  |  | Multivariate |  |
|  | p-value | OR | 95% CI | p-value | OR | 95% CI |
| **Pre-BCPS** |  |  |  |  |  |  |
| PAI | **0.004** | 0.994 | 0.990-0.998 |  |  |  |
| Right PAI | **0.002** | 0.990 | 0.983-0.996 | **0.002** | 0.989 | 0.982-0.996 |
| Left PAI | 0.108 | 0.994 | 0.986-1.001 |  |  |  |
| Left-to-right ratio | 0.148 | 1.453 | 0.876-2.409 |  |  |  |
| Symmetry index | **0.066** | 3.388 | 0.925-12.414 |  |  |  |
| Hemoglobin | 0.215 | 0.920 | 0.807-1.049 |  |  |  |
| PAP | 0.224 | 1.046 | 0.973-1.124 |  |  |  |
| TPG | 0.491 | 1.028 | 0.951-1.111 |  |  |  |
| LAP | 0.257 | 1.059 | 0.959-1.169 |  |  |  |
| EDP | 0.058 | 1.095 | 0.997-1.202 |  |  |  |
| SO_2_ | 0.571 | 0.991 | 0.962-1.022 |  |  |  |
| Ventricular dysfunction | 0.642 | 1.211 | 0.540-2.716 |  |  |  |
| Significant AVVR | 0.916 | 1.012 | 0.805-1.274 |  |  |  |
| **Pre-TCPC** |  |  |  |  |  |  |
| PAI | 0.244 | 0.997 | 0.993-1.002 |  |  |  |
| Right PAI | 0.606 | 0.998 | 0.993-1.004 |  |  |  |
| Left PAI | 0.153 | 0.994 | 0.985-1.002 |  |  |  |
| Left-to-right ratio | 0.524 | 0.777 | 0.357-1.688 |  |  |  |
| Symmetry index | 0.921 | 0.946 | 0.315-2.842 |  |  |  |
| Hemoglobin | 0.499 | 1.050 | 0.912-1.208 |  |  |  |
| PAP | 0.327 | 1.056 | 0.947-1.177 |  |  |  |
| TPG | 0.716 | 0.969 | 0.817-1-149 |  |  |  |
| LAP | 0.273 | 1.069 | 0.949-1.204 |  |  |  |
| EDP | 0.210 | 1.072 | 0.962-1.196 |  |  |  |
| SO_2_ | 0.365 | 1.026 | 0.971-1.083 |  |  |  |
| Ventricular dysfunction | 0.570 | 1.212 | 0.625-2.351 |  |  |  |
| Significant AVVR | 0.421 | 1.099 | 0.873-1.385 |  |  |  |

| Table E7c. Impact of PAI on chylothorax after TCPC (other types of BCPS patients) | | | | | | |
| --- | --- | --- | --- | --- | --- | --- |
| Variables |  | Univariate |  |  | Multivariate |  |
|  | p-value | OR | 95% CI | p-value | OR | 95% CI |
| **Pre-BCPS** |  |  |  |  |  |  |
| PAI | 0.740 | 0.999 | 0.995-1.004 |  |  |  |
| Right PAI | 0.439 | 0.997 | 0.990-1.004 |  |  |  |
| Left PAI | 0.707 | 1.002 | 0.993-1.010 |  |  |  |
| Left-to-right ratio | 0.683 | 1.188 | 0.519-2.718 |  |  |  |
| Symmetry index | 0.069 | 13.080 | 0.820-208.668 |  |  |  |
| Hemoglobin | 0.343 | 0.875 | 0.663-1.154 |  |  |  |
| PAP | 0.948 | 0.995 | 0.866-1.144 |  |  |  |
| TPG | 0.170 | 0.876 | 0.725-1.058 |  |  |  |
| LAP | 0.961 | 1.004 | 0.855-1.179 |  |  |  |
| EDP | 0.633 | 0.958 | 0.801-1.144 |  |  |  |
| SO_2_ | 0.071 | 1.093 | 0.992-1.204 |  |  |  |
| Ventricular dysfunction | . | . | . |  |  |  |
| Significant AVVR | 0.138 | 0.604 | 0.311-1.176 |  |  |  |
| **Pre-TCPC** |  |  |  |  |  |  |
| PAI | 0.170 | 1.006 | 0.998-1.014 |  |  |  |
| Right PAI | 0.105 | 1.009 | 0.998-1.021 |  |  |  |
| Left PAI | 0.812 | 1.002 | 0.989-1.015 |  |  |  |
| Left-to-right ratio | 0.136 | 0.439 | 0.149-1.297 |  |  |  |
| Symmetry index | 0.988 | 1.019 | 0.082-12.698 |  |  |  |
| Hemoglobin | 0.375 | 0.888 | 0.684-1.154 |  |  |  |
| PAP | 0.149 | 1.170 | 0.945-1.448 |  |  |  |
| TPG | 0.524 | 1.128 | 0.780-1.630 |  |  |  |
| LAP | 0.160 | 1.211 | 0.927-1.581 |  |  |  |
| EDP | 0.184 | 1.176 | 0.925-1.495 |  |  |  |
| SO_2_ | 0.900 | 0.993 | 0.884-1.115 |  |  |  |
| Ventricular dysfunction | 1.000 | 0.000 |  |  |  |  |
| Significant AVVR | 0.923 | 1.026 | 0.617-1.706 |  |  |  |

**Supplementary Table E8**

Impact of PAI on ascites after TCPC

| Table E8a. Impact of PAI on ascites after TCPC (all patients) | | | | |  |  |
| --- | --- | --- | --- | --- | --- | --- |
| Variables |  | Univariate |  |  | Multivariate |  |
|  | p-value | OR | 95% CI | p-value | OR | 95% CI |
| **Pre-BCPS** |  |  |  |  |  |  |
| PAI | **0.035** | 0.996 | 0.992-1.000 |  |  |  |
| Right PAI | **0.025** | 0.993 | 0.987-0.999 | **0.024** | 0.993 | 0.986-0.999 |
| Left PAI | 0.213 | 0.996 | 0.989-1.002 |  |  |  |
| Left-to-right ratio | 0.990 | 0.997 | 0.587-1.693 |  |  |  |
| Symmetry index | 0.393 | 1.780 | 0.474-6.677 |  |  |  |
| Hemoglobin | 0.221 | 1.087 | 0.951-1.243 |  |  |  |
| PAP | 0.926 | 1.004 | 0.931-1.082 |  |  |  |
| TPG | 0.728 | 0.986 | 0.909-1.069 |  |  |  |
| LAP | 0.984 | 0.999 | 0.907-1.101 |  |  |  |
| EDP | 0.418 | 0.961 | 0.872-1.058 |  |  |  |
| SO_2_ | 0.696 | 1.007 | 0.974-1.040 |  |  |  |
| Ventricular dysfunction | **0.048** | 2.251 | 1.008-5.029 | **0.033** | 2.442 | 1.077-5.539 |
| Significant AVVR | 0.115 | 1.215 | 0.954-1.547 |  |  |  |
| **Pre-TCPC** |  |  |  |  |  |  |
| PAI | **0.015** | 0.994 | 0.989-0.999 | **0.005** | 0.990 | 0.982-0.997 |
| Right PAI | **0.004** | 0.989 | 0.982-0.996 |  |  |  |
| Left PAI | 0.420 | 0.997 | 0.989-1.005 |  |  |  |
| Left-to-right ratio | 0.015 | 1.977 | 1.139-3.429 |  |  |  |
| Symmetry index | 0.934 | 0.953 | 0.303-3.002 |  |  |  |
| Hemoglobin | 0.651 | 0.968 | 0.841-1.115 |  |  |  |
| PAP | **0.007** | 1.162 | 1.042-1.296 | **0.008** | 1.172 | 1.043-1.318 |
| TPG | 0.085 | 1.156 | 0.980-1.364 |  |  |  |
| LAP | 0.137 | 1.097 | 0.971-1.238 |  |  |  |
| EDP | 0.319 | 1.059 | 0.947-1.184 |  |  |  |
| SO_2_ | 0.091 | 0.956 | 0.908-1.007 |  |  |  |
| Ventricular dysfunction | 0.440 | 1.315 | 0.656-2.633 |  |  |  |
| Significant AVVR | 0.098 | 1.223 | 0.963-1.551 |  |  |  |

| Table E8b. Impact of PAI on ascites after TCPC (right-sided BCPS patients) | | | | | |  |
| --- | --- | --- | --- | --- | --- | --- |
| Variables |  | Univariate |  |  | Multivariate |  |
|  | p-value | OR | 95% CI | p-value | OR | 95% CI |
| **Pre-BCPS** |  |  |  |  |  |  |
| PAI | **0.007** | 0.992 | 0.986-0.998 | **0.005** | 0.991 | 0.985-0.997 |
| Right PAI | **0.015** | 0.989 | 0.980-0.998 |  |  |  |
| Left PAI | **0.031** | 0.988 | 0.978-0.999 |  |  |  |
| Left-to-right ratio | 0.775 | 0.906 | 0.462-1.777 |  |  |  |
| Symmetry index | 0.663 | 1.411 | 0.300-6.646 |  |  |  |
| Hemoglobin | 0.275 | 1.090 | 0.934-1.272 |  |  |  |
| PAP | 0.177 | 0.936 | 0.851-1.030 |  |  |  |
| TPG | 0.167 | 0.930 | 0.839-1.031 |  |  |  |
| LAP | 0.913 | 1.007 | 0.895-1.132 |  |  |  |
| EDP | 0.940 | 0.996 | 0.890-1.114 |  |  |  |
| SO_2_ | 0.628 | 1.010 | 0.969-1.053 |  |  |  |
| Ventricular dysfunction | **0.031** | 2.459 | 1.087-5.561 | **0.016** | 2.769 | 1.206-6.356 |
| Significant AVVR | 0.331 | 1.143 | 0.730-1.497 |  |  |  |
| **Pre-TCPC** |  |  |  |  |  |  |
| PAI | **0.011** | 0.992 | 0.986-0.998 | **0.017** | 0.993 | 0.986-0.999 |
| Right PAI | **0.024** | 0.990 | 0.982-0.999 |  |  |  |
| Left PAI | 0.051 | 0.989 | 0.978-1.000 |  |  |  |
| Left-to-right ratio | 0.367 | 1.473 | 0.635-3.415 |  |  |  |
| Symmetry index | 0.629 | 0.722 | 0.192-2.708 |  |  |  |
| Hemoglobin | 0.202 | 0.896 | 0.758-1.060 |  |  |  |
| PAP | **0.006** | 1.199 | 1.053-1.365 | **0.015** | 1.179 | 1.033-1.347 |
| TPG | 0.108 | 1.164 | 0.967-1.401 |  |  |  |
| LAP | 0.102 | 1.122 | 0.978-1.288 |  |  |  |
| EDP | 0.118 | 1.108 | 0.974-1.259 |  |  |  |
| SO_2_ | 0.200 | 0.962 | 0.908-1.020 |  |  |  |
| Ventricular dysfunction | 0.547 | 1.258 | 0.597-2.653 |  |  |  |
| Significant AVVR | 0.196 | 1.199 | 0.911-1.578 |  |  |  |

| Table E8c. Impact of PAI on ascites after TCPC (other types of BCPS patients) | | | | | |  |
| --- | --- | --- | --- | --- | --- | --- |
| Variables |  | Univariate |  |  | Multivariate |  |
|  | p-value | OR | 95% CI | p-value | OR | 95% CI |
| **Pre-BCPS** |  |  |  |  |  |  |
| PAI | 0.326 | 0.997 | 0.992-1.003 |  |  |  |
| Right PAI | 0.243 | 0.995 | 0.986-1.004 |  |  |  |
| Left PAI | 0.703 | 0.998 | 0.989-1.007 |  |  |  |
| Left-to-right ratio | 0.955 | 0.975 | 0.401-2.369 |  |  |  |
| Symmetry index | 0.432 | 2.738 | 0.222-33.820 |  |  |  |
| Hemoglobin | 0.658 | 1.064 | 0.809-1.399 |  |  |  |
| PAP | **0.030** | 1.209 | 1.018-1.435 | **0.030** | 1.209 | 1.018-1.435 |
| TPG | 0.118 | 1.144 | 0.956-1.354 |  |  |  |
| LAP | 0.779 | 0.976 | 0.821-1.159 |  |  |  |
| EDP | 0.228 | 0.890 | 0.737-1.075 |  |  |  |
| SO_2_ | 0.828 | 0.995 | 0.951-1.041 |  |  |  |
| Ventricular dysfunction | . | . | . |  |  |  |
| Significant AVVR | 0.083 | 1.794 | 0.927-3.474 |  |  |  |
| **Pre-TCPC** |  |  |  |  |  |  |
| PAI | 0.385 | 0.996 | 0.988-1.005 |  |  |  |
| Right PAI | 0.093 | 0.988 | 0.974-1.002 |  |  |  |
| Left PAI | 0.650 | 1.003 | 0.990-1.017 |  |  |  |
| Left-to-right ratio | 0.100 | 2.083 | 0.870-4.989 |  |  |  |
| Symmetry index | 0.803 | 1.396 | 0.101-19.246 |  |  |  |
| Hemoglobin | 0.387 | 1.121 | 0.865-1.453 |  |  |  |
| PAP | 0.591 | 1.059 | 0.960-1.303 |  |  |  |
| TPG | 0.303 | 1.228 | 0.831-1.813 |  |  |  |
| LAP | 0.883 | 0.980 | 0.747-1.285 |  |  |  |
| EDP | 0.355 | 0.895 | 0.709-1.131 |  |  |  |
| SO_2_ | 0.133 | 0.909 | 0.802-1.030 |  |  |  |
| Ventricular dysfunction | 1.000 |  |  |  |  |  |
| Significant AVVR | 0.383 | 1.254 | 0.754-2.085 |  |  |  |

**Supplementary Table E9**

Impact of PAI on survival after TCPC

| Table E9a. Impact of PAI on survival after TCPC (all patients) | | | | | |  |  |
| --- | --- | --- | --- | --- | --- | --- | --- |
| Variables | Univariate | | |  | Multivariate | | |
|  | p-value | HR | 95% CI |  | p-value | HR | 95% CI |
| **Pre-BCPS** |  |  |  |  |  |  |  |
| PAI | 0.629 | 1.002 | 0.993-1.011 |  |  |  |  |
| Right PAI | 0.815 | 1.002 | 0.988-1.016 |  |  |  |  |
| Left PAI | 0.525 | 1.005 | 0.989-1.022 |  |  |  |  |
| Left-to-right ratio | 0.598 | 0.536 | 0.053-5.445 |  |  |  |  |
| Symmetry index | 0.777 | 1.935 | 0.020-185.602 |  |  |  |  |
| Hemoglobin | 0.363 | 1.208 | 0.804-1.816 |  |  |  |  |
| PAP | **0.046** | 1.223 | 1.004-1.491 |  | **0.032** | 1.257 | 1.019-1.551 |
| TPG | 0.086 | 1.194 | 0.975-1.461 |  |  |  |  |
| LAP | 0.936 | 0.987 | 0.721-1.352 |  |  |  |  |
| EDP | 0.500 | 0.899 | 0.659-1.226 |  |  |  |  |
| SO_2_ | **0.026** | 1.194 | 1.021-1.395 |  | **0.029** | 1.286 | 1.025-1.613 |
| Ventricular dysfunction | 0.808 | 0.130 | 0.000-1775730 |  |  |  |  |
| Significant AVVR | 0.473 | 0.712 | 0.281-1.800 |  |  |  |  |
| **Pre-TCPC** |  |  |  |  |  |  |  |
| PAI | 0.522 | 0.995 | 0.981-1.010 |  |  |  |  |
| Right PAI | 0.129 | 0.981 | 0.956-1.006 |  |  |  |  |
| Left PAI | 0.483 | 1.007 | 0.988-1.026 |  |  |  |  |
| Left-to-right ratio | 0.120 | 2.447 | 0.791-7.569 |  |  |  |  |
| Symmetry index | **0.032** | 12.930 | 1.238-133.023 |  | **0.032** | 12.930 | 1.238-133.023 |
| Hemoglobin | 0.133 | 1.327 | 0.918-1.920 |  |  |  |  |
| PAP | 0.873 | 1.028 | 0.735-1.438 |  |  |  |  |
| TPG | 0.276 | 1.275 | 0.824-1.973 |  |  |  |  |
| LAP | 0.519 | 0.878 | 0.592-1.303 |  |  |  |  |
| EDP | 0.835 | 0.965 | 0.689-1.352 |  |  |  |  |
| SO_2_ | 0.270 | 0.933 | 0.826-1.055 |  |  |  |  |
| Ventricular dysfunction | 0.799 | 0.169 | 0.000-41553.3 |  |  |  |  |
| Significant AVVR | 0.818 | 0.918 | 0.443-1.902 |  |  |  |  |
| **Post-TCPC** |  |  |  |  |  |  |  |
| PAI | 0.571 | 1.006 | 0.986-1.026 |  |  |  |  |
| Right PAI | 0.724 | 1.005 | 0.978-1.032 |  |  |  |  |
| Left PAI | 0.550 | 1.012 | 0.974-1.051 |  |  |  |  |
| Left-to-right ratio | 0.970 | 0.917 | 0.010-84.880 |  |  |  |  |
| Symmetry index | 0.758 | 2.881 | 0.003-2432.262 |  |  |  |  |

| Table E9b. Impact of PAI on survival after TCPC (right-sided BCPS patients) | | | | | | |  |
| --- | --- | --- | --- | --- | --- | --- | --- |
| Variables |  | Univariate |  |  |  | Multivariate |  |
|  | p-value | HR | 95% CI |  | p-value | HR | 95% CI |
| **Pre-BCPS** |  |  |  |  |  |  |  |
| PAI | 0.384 | 0.986 | 0.954-1.018 |  |  |  |  |
| Right PAI | 0.646 | 0.992 | 0.956-1.028 |  |  |  |  |
| Left PAI | 0.229 | 0.955 | 0.887-1.029 |  |  |  |  |
| Left-to-right ratio | 0.286 | 0.071 | 0.001-9.176 |  |  |  |  |
| Symmetry index | 0.420 | 0.081 | 0.000-36.054 |  |  |  |  |
| Hemoglobin | 0.367 | 1.275 | 0.752-2.163 |  |  |  |  |
| PAP | 0.545 | 1.129 | 0.762-1.672 |  |  |  |  |
| TPG | 0.773 | 1.069 | 0.679-1.682 |  |  |  |  |
| LAP | 0.677 | 0.905 | 0.567-1.446 |  |  |  |  |
| EDP | 0.687 | 0.918 | 0.607-1.390 |  |  |  |  |
| SO_2_ | 0.389 | 1.080 | 0.907-1.286 |  |  |  |  |
| Ventricular dysfunction | 0.840 | 0.128 | 0.000-64331254 |  |  |  |  |
| Significant AVVR | 0.462 | 0.618 | 0.171-2.231 |  |  |  |  |
| **Pre-TCPC** |  |  |  |  |  |  |  |
| PAI | 0.225 | 0.982 | 0.954-1.011 |  |  |  |  |
| Right PAI | 0.062 | 0.956 | 0.911-1.002 |  |  |  |  |
| Left PAI | 0.925 | 1.002 | 0.969-1.035 |  |  |  |  |
| Left-to-right ratio | 0.058 | 8.373 | 0.939-43.281 |  |  |  |  |
| Symmetry index | 0.130 | 10.511 | 0.500-221.142 |  |  |  |  |
| Hemoglobin | 0.676 | 0.882 | 0.489-1.591 |  |  |  |  |
| PAP | 0.334 | 0.761 | 0.438-1.323 |  |  |  |  |
| TPG | 0.852 | 0.930 | 0.436-1.983 |  |  |  |  |
| LAP | 0.272 | 0.700 | 0.370-1.323 |  |  |  |  |
| EDP | 0.679 | 1.100 | 0.702-1.723 |  |  |  |  |
| SO_2_ | 0.651 | 0.958 | 0.793-1.156 |  |  |  |  |
| Ventricular dysfunction | 0.837 | 0.179 | 0.000-2396458.8 |  |  |  |  |
| Significant AVVR | 0.679 | 0.796 | 0.269-2.354 |  |  |  |  |
| **Post-TCPC** |  |  |  |  |  |  |  |
| PAI | 0.311 | 1.011 | 0.988-1.035 |  |  |  |  |
| Right PAI | 0.544 | 1.009 | 0.980-1.040 |  |  |  |  |
| Left PAI | 0.240 | 1.034 | 0.978-1.093 |  |  |  |  |
| Left-to-right ratio | 0.807 | 2.273 | 0.003-1626.759 |  |  |  |  |
| Symmetry index | 0.668 | 8.343 | 0.001-13404.7 |  |  |  |  |

| Table E9c. Impact of PAI on survival after TCPC (other types of BCPS patients) | | | | | | | |
| --- | --- | --- | --- | --- | --- | --- | --- |
| Variables |  | Univariate |  |  |  | Multivariate |  |
|  | p-value | HR | 95% CI |  | p-value | HR | 95% CI |
| **Pre-BCPS** |  |  |  |  |  |  |  |
| PAI | 0.579 | 1.003 | 0.993-1.012 |  |  |  |  |
| Right PAI | 0.836 | 1.001 | 0.987-1.016 |  |  |  |  |
| Left PAI | 0.415 | 1.007 | 0.990-1.025 |  |  |  |  |
| Left-to-right ratio | 0.972 | 0.897 | 0.103-7.821 |  |  |  |  |
| Symmetry index | 0.366 | 130.180 | 0.003-5052221 |  |  |  |  |
| Hemoglobin | 0.734 | 1.117 | 0.590-2.113 |  |  |  |  |
| PAP | 0.067 | 1.216 | 0.986-1.498 |  |  |  |  |
| TPG | 0.109 | 1.235 | 0.954-1.599 |  |  |  |  |
| LAP | 0.809 | 1.038 | 0.767-1.404 |  |  |  |  |
| EDP | 0.677 | 0.914 | 0.600-1.394 |  |  |  |  |
| SO_2_ | 0.083 | 1.523 | 0.946-2.451 |  |  |  |  |
| Ventricular dysfunction | . | . | . |  |  |  |  |
| Significant AVVR | 0.811 | 0.830 | 0.180-3.818 |  |  |  |  |
| **Pre-TCPC** |  |  |  |  |  |  |  |
| PAI | 0.996 | 1.000 | 0.984-1.017 |  |  |  |  |
| Right PAI | 0.960 | 0.999 | 0.974-1.025 |  |  |  |  |
| Left PAI | 0.973 | 1.000 | 0.974-1.028 |  |  |  |  |
| Left-to-right ratio | 0.711 | 0.668 | 0.079-5.636 |  |  |  |  |
| Symmetry index | 0.409 | 12.291 | 0.032-4749.390 |  |  |  |  |
| Hemoglobin | 0.062 | 1.442 | 0.982-2.119 |  |  |  |  |
| PAP | 0.315 | 1.203 | 0.839-1.725 |  |  |  |  |
| TPG | 0.059 | 2.105 | 0.971-4.560 |  |  |  |  |
| LAP | 0.899 | 0.966 | 0.569-1.641 |  |  |  |  |
| EDP | 0.285 | 0.777 | 0.490-1.233 |  |  |  |  |
| SO_2_ | 0.120 | 0.865 | 0.720-1.039 |  |  |  |  |
| Ventricular dysfunction | 0.899 | 0.048 | 0.000-9.51E+18 |  |  |  |  |
| Significant AVVR | 0.961 | 0.976 | 0.365-2.605 |  |  |  |  |
| **Post-TCPC** |  |  |  |  |  |  |  |
| PAI | 0.768 | 0.995 | 0.963-1.028 |  |  |  |  |
| Right PAI | 0.994 | 1.000 | 0.948-1.055 |  |  |  |  |
| Left PAI | 0.636 | 0.986 | 0.928-1.047 |  |  |  |  |
| Left-to-right ratio | 0.547 | 0.171 | 0.001-53.719 |  |  |  |  |
| Symmetry index | 0.732 | 0.245 | 0-757.252 |  |  |  |  |

**Supplementary Table E10**

Impact of PAI on failing Fontan after TCPC

| Table E10a. Impact of PAI on failing Fontan after TCPC (all patients) | | | | | | |  |
| --- | --- | --- | --- | --- | --- | --- | --- |
| Variables | Univariate | | |  | Multivariate | | |
|  | p-value | HR | 95% CI |  | p-value | HR | 95% CI |
| **Pre-BCPS** |  |  |  |  |  |  |  |
| PAI | 0.596 | 0.999 | 0.994-1.003 |  |  |  |  |
| Right PAI | 0.748 | 0.999 | 0.991-1.006 |  |  |  |  |
| Left PAI | 0.493 | 0.997 | 0.989-1.005 |  |  |  |  |
| Left-to-right ratio | 0.990 | 0.996 | 0.541-1.835 |  |  |  |  |
| Symmetry index | 0.845 | 0.851 | 0.171-4.250 |  |  |  |  |
| Hemoglobin | 0.474 | 0.933 | 0.772-1.128 |  |  |  |  |
| PAP | 0.369 | 1.042 | 0.953-1.138 |  |  |  |  |
| TPG | 0.881 | 1.008 | 0.907-1.121 |  |  |  |  |
| LAP | 0.392 | 1.060 | 0.927-1.212 |  |  |  |  |
| EDP | 0.192 | 1.080 | 0.962-1.212 |  |  |  |  |
| SO_2_ | 0.885 | 0.997 | 0.961-1.035 |  |  |  |  |
| Ventricular dysfunction | 0.905 | 1.102 | 0.223-5.451 |  |  |  |  |
| Significant AVVR | 0.522 | 0.891 | 0.625-1.269 |  |  |  |  |
| **Pre-TCPC** |  |  |  |  |  |  |  |
| PAI | 0.311 | 0.997 | 0.991-1.003 |  |  |  |  |
| Right PAI | 0.874 | 0.999 | 0.991-1.007 |  |  |  |  |
| Left PAI | 0.186 | 0.992 | 0.981-1.004 |  |  |  |  |
| Left-to-right ratio | 0.347 | 0.642 | 0.255-1.616 |  |  |  |  |
| Symmetry index | 0.550 | 0.631 | 0.140-2.853 |  |  |  |  |
| Hemoglobin | 0.063 | 1.179 | 0.991-1.403 |  |  |  |  |
| PAP | **<0.001** | 1.229 | 1.090-1.385 |  | **0.020** | 1.202 | 1.030-1.403 |
| TPG | **0.003** | 1.351 | 1.111-1.642 |  |  |  |  |
| LAP | 0.183 | 1.109 | 0.952-1.291 |  |  |  |  |
| EDP | 0.663 | 1.032 | 0.897-1.187 |  |  |  |  |
| SO_2_ | 0.147 | 0.957 | 0.901-1.016 |  |  |  |  |
| Ventricular dysfunction | 0.404 | 0.103 | 0.000-21.571 |  |  |  |  |
| Significant AVVR | 0.807 | 0.961 | 0.701-1.319 |  |  |  |  |
| **Post-TCPC** |  |  |  |  |  |  |  |
| PAI | 0.880 | 1.001 | 0.993-1.008 |  |  |  |  |
| Right PAI | 0.379 | 0.995 | 0.985-1.006 |  |  |  |  |
| Left PAI | 0.121 | 1.009 | 0.998-1.020 |  |  |  |  |
| Left-to-right ratio | 0.321 | 1.660 | 0.611-4.511 |  |  |  |  |
| Symmetry index | 0.178 | 3.133 | 0.596-16.483 |  |  |  |  |

| Table E10b. Impact of PAI on failing Fontan after TCPC (right-sided BCPS patients) | | | | | | | |
| --- | --- | --- | --- | --- | --- | --- | --- |
| Variables | Univariate | | |  | Multivariate | | |
|  | p-value | HR | 95% CI |  | p-value | HR | 95% CI |
| **Pre-BCPS** |  |  |  |  |  |  |  |
| PAI | 0.837 | 1.001 | 0.995-1.006 |  |  |  |  |
| Right PAI | 0.584 | 1.002 | 0.994-1.010 |  |  |  |  |
| Left PAI | 0.700 | 0.998 | 0.987-1.009 |  |  |  |  |
| Left-to-right ratio | 0.866 | 0.933 | 0.418-2.084 |  |  |  |  |
| Symmetry index | 0.508 | 0.536 | 0.084-3.403 |  |  |  |  |
| Hemoglobin | 0.284 | 0.890 | 0.718-1.102 |  |  |  |  |
| PAP | 0.315 | 1.051 | 0.953-1.159 |  |  |  |  |
| TPG | 0.559 | 1.034 | 0.925-1.156 |  |  |  |  |
| LAP | 0.805 | 1.020 | 0.871-1.195 |  |  |  |  |
| EDP | 0.556 | 1.040 | 0.914-1.183 |  |  |  |  |
| SO_2_ | 0.673 | 0.991 | 0.951-1.033 |  |  |  |  |
| Ventricular dysfunction | 0.943 | 1.060 | 0.214-5.249 |  |  |  |  |
| Significant AVVR | 0.362 | 0.833 | 0.563-1.233 |  |  |  |  |
| **Pre-TCPC** |  |  |  |  |  |  |  |
| PAI | 0.138 | 0.994 | 0.987-1.002 |  |  |  |  |
| Right PAI | 0.571 | 0.997 | 0.988-1.007 |  |  |  |  |
| Left PAI | When | 0.984 | 0.968-1.001 |  |  |  |  |
| Left-to-right ratio | 0.565 | 0.693 | 0.199-2.412 |  |  |  |  |
| Symmetry index | 0.113 | 0.231 | 0.038-1.411 |  |  |  |  |
| Hemoglobin | 0.425 | 1.085 | 0.888-1.327 |  |  |  |  |
| PAP | **<0.001** | 1.304 | 1.119-1.520 |  | **<0.001** | 1.311 | 1.123-1.531 |
| TPG | **0.007** | 1.327 | 1.080-1.630 |  |  |  |  |
| LAP | 0.079 | 1.167 | 0.982-1.387 |  |  |  |  |
| EDP | 0.281 | 1.094 | 0.929-1.287 |  |  |  |  |
| SO_2_ | 0.213 | 0.960 | 0.901-1.024 |  |  |  |  |
| Ventricular dysfunction |  |  |  |  |  |  |  |
| Significant AVVR |  |  |  |  |  |  |  |
| **Post-TCPC** |  |  |  |  |  |  |  |
| PAI | 0.705 | 0.998 | 0.990-1.007 |  |  |  |  |
| Right PAI | 0.301 | 0.994 | 0.982-1.006 |  |  |  |  |
| Left PAI | 0.201 | 1.012 | 0.994-1.031 |  |  |  |  |
| Left-to-right ratio | 0.376 | 1.883 | 0.464-7.647 |  |  |  |  |
| Symmetry index | 0.226 | 3.357 | 0.472-23.884 |  |  |  |  |

| Table E10c. Impact of PAI on failing Fontan after TCPC (other types of BCPS patients) | | | | | | | |
| --- | --- | --- | --- | --- | --- | --- | --- |
| Variables | Univariate | | |  | Multivariate | | |
|  | p-value | HR | 95% CI |  | p-value | HR | 95% CI |
| **Pre-BCPS** |  |  |  |  |  |  |  |
| PAI | 0.237 | 0.993 | 0.981-1.005 |  |  |  |  |
| Right PAI | 0.163 | 0.984 | 0.961-1.007 |  |  |  |  |
| Left PAI | 0.497 | 0.995 | 0.981-1.010 |  |  |  |  |
| Left-to-right ratio | 0.872 | 1.084 | 0.407-2.887 |  |  |  |  |
| Symmetry index | 0.514 | 3.076 | 0.105-90.201 |  |  |  |  |
| Hemoglobin | 0.617 | 1.111 | 0.735-1.681 |  |  |  |  |
| PAP | 0.960 | 1.007 | 0.756-1.342 |  |  |  |  |
| TPG | 0.325 | 0.787 | 0.488-1.268 |  |  |  |  |
| LAP | 0.129 | 1.174 | 0.954-1.443 |  |  |  |  |
| EDP | 0.092 | 1.324 | 0.955-1.836 |  |  |  |  |
| SO_2_ | 0.733 | 1.018 | 0.920-1.125 |  |  |  |  |
| Ventricular dysfunction | . | . | . |  |  |  |  |
| Significant AVVR | 0.445 | 1.449 | 0.559-3.757 |  |  |  |  |
| **Pre-TCPC** |  |  |  |  |  |  |  |
| PAI | 0.371 | 1.005 | 0.994-1.017 |  |  |  |  |
| Right PAI | 0.355 | 1.010 | 0.989-1.031 |  |  |  |  |
| Left PAI | 0.583 | 1.005 | 0.987-1.023 |  |  |  |  |
| Left-to-right ratio | 0.503 | 0.566 | 0.107-2.995 |  |  |  |  |
| Symmetry index | 0.081 | 860.917 | 0.433-1713568 |  |  |  |  |
| Hemoglobin | **0.011** | 1.533 | 1.101-2.135 |  | **0.011** | 1.533 | 1.101-2.135 |
| PAP | 0.426 | 1.121 | 0.846-1.485 |  |  |  |  |
| TPG | 0.263 | 1.381 | 0.785-2.429 |  |  |  |  |
| LAP | 0.708 | 0.922 | 0.604-1.409 |  |  |  |  |
| EDP | 0.225 | 0.811 | 0.578-1.138 |  |  |  |  |
| SO_2_ | 0.414 | 0.922 | 0.758-1.121 |  |  |  |  |
| Ventricular dysfunction | 0.705 | 0.039 | 0.000-791660 |  |  |  |  |
| Significant AVVR | 0.957 | 1.019 | 0.506-2.054 |  |  |  |  |
| **Post-TCPC** |  |  |  |  |  |  |  |
| PAI | 0.259 | 1.010 | 0.992-1.029 |  |  |  |  |
| Right PAI | 0.993 | 1.000 | 0.971-1.030 |  |  |  |  |
| Left PAI | 0.139 | 1.016 | 0.995-1.038 |  |  |  |  |
| Left-to-right ratio | 0.266 | 3.490 | 0.386-31.564 |  |  |  |  |
| Symmetry index | 0.374 | 7.700 | 0.086-690.180 |  |  |  |  |

**Supplementary Table E11**

Impact of PAI on exercise capacity after TCPC

| Table E11a. Impact of PAI on exercise capacity after TCPC (all patients) | | | | | |
| --- | --- | --- | --- | --- | --- |
| Variables | Peak VO2 | |  | % of normal | |
|  | p-value | Pearson |  | p-value | Pearson |
| **Pre-BCPS** |  |  |  |  |  |
| PAI | 0.251 | 0.295 |  | 0.959 | 0.014 |
| Right PAI | 0.354 | 0.240 |  | 0.724 | 0.092 |
| Left PAI | 0.422 | 0.208 |  | 0.806 | 0.064* |
| Left-to-right ratio | 0.716 | 0.095 |  | 0.581 | 0.144* |
| Symmetry index | 0.586 | 0.142 |  | 0.953 | 0.015* |
| Hemoglobin | 0.226 | 0.263* |  | 0.130 | 0.325* |
| PAP | 0.787 | 0.083 |  | 0.424 | 0.243* |
| TPG | 0.444 | 0.233 |  | 0.673 | 0.129* |
| LAP | 0.566 | 0.123* |  | 0.383 | 0.187* |
| EDP | 0.077 | 0.376 |  | 0.625 | 0.108 |
| SO_2_ | 0.865 | 0.037 |  | 0.448 | 0.162* |
| **Pre-TCPC** |  |  |  |  |  |
| PAI | 0.487 | 0.143* |  | 0.815 | 0.048* |
| Right PAI | 0.522 | 0.131* |  | 0.732 | 0.071 |
| Left PAI | 0.721 | 0.074* |  | 0.521 | 0.132* |
| Left-to-right ratio | 0.549 | 0.123 |  | 0.659 | 0.091* |
| Symmetry index | 0.802 | 0.052 |  | 0.461 | 0.151* |
| Hemoglobin | 0.765 | 0.059 |  | 0.880 | 0.170* |
| PAP | **0.011** | 0.471* |  | 0.388 | 0.193* |
| TPG | 0.477 | 0.140* |  | 0.324 | 0.013* |
| LAP | 0.136 | 0.289* |  | 0.948 | 0.008 |
| EDP | 0.077 | 0.346* |  | 0.967 | 0.003* |
| SO_2_ | 0.491 | 0.138 |  | 0.987 | 0.048* |
| **Post-TCPC** |  |  |  |  |  |
| PAI | 0.601 | 0.132 |  | 0.345 | 0.236 |
| Right PAI | 0.547 | 0.152 |  | 0.277 | 0.271 |
| Left PAI | 0.961 | 0.012 |  | 0.925 | 0.024 |
| Left-to-right ratio | 0.949 | 0.016* |  | .0.809 | 0.061* |
| Symmetry index | 0.827 | 0.055 |  | 0.876 | 0.040* |
| *: negative value |  |  |  |  |  |

| Table E11b. Impact of PAI on exercise capacity after TCPC (right side BCPS patients) | | | | | |
| --- | --- | --- | --- | --- | --- |
| Variables | Peak VO2 | |  | % of normal | |
|  | p-value | Pearson |  | p-value | Pearson |
| **Pre-BCPS** |  |  |  |  |  |
| PAI | 0.429 | 0.302 |  | 0.819 | 0.089* |
| Right PAI | 0.446 | 0.292 |  | 0.983 | 0.008* |
| Left PAI | 0.779 | 0.109 |  | 0.567 | 0.221* |
| Left-to-right ratio | 0.519 | 0.249* |  | 0.597 | 0.205* |
| Symmetry index | 0.768 | 0.115* |  | 0.874 | 0.062 |
| Hemoglobin | .0540 | 0.172* |  | 0.225 | 0.333* |
| PAP | 0.800 | 0.107 |  | 0.808 | 0.103* |
| TPG | 0.519 | 0.269 |  | 0.912 | 0.047 |
| LAP | 0.195 | 0.342* |  | 0.428 | 0.216* |
| EDP | 0.273 | 0.302 |  | 0.289 | 0.293 |
| SO_2_ | 0.746 | 0.088 |  | 0.249 | 0.306* |
| **Pre-TCPC** |  |  |  |  |  |
| PAI | 0.274 | 0.291 |  | 0.417 | 0.218 |
| Right PAI | 0.300 | 0.277 |  | 0.299 | 0.277 |
| Left PAI | 0.770 | 0.079 |  | 0.654 | 0.122* |
| Left-to-right ratio | 0.848 | 0.052* |  | 0.680 | 0.112* |
| Symmetry index | 0.633 | 0.129* |  | 0.542 | 0.165* |
| Hemoglobin | 0.776 | 0.072 |  | 0.269 | 0.275 |
| PAP | **0.038** | 0.493* |  | 0.46.691 | 0.184* |
| TPG | 0.249 | 0.287 |  | 0.725 | 0.101* |
| LAP | 0.186 | 0.327 |  | 0.176 | 0.089* |
| EDP | 0.112 | 0.399 |  | 0.673 | 0.377* |
| SO_2_ | 0.337 | 0.248 |  | 0.417 | 0.110* |
| **Post-TCPC** |  |  |  |  |  |
| PAI | 0.060 | 0.556 |  | 0.263 | 0.351 |
| Right PAI | 0.052 | 0.571 |  | 0.301 | 0.326 |
| Left PAI | 0.452 | 0.240* |  | 0.945 | 0.022* |
| Left-to-right ratio | **0.040** | 0.599* |  | 0.476 | 0.228* |
| Symmetry index | **0.040** | 0.599* |  | .0.476 | 0.228* |
| *: negative value |  |  |  |  |  |

| Table E11c. Impact of PAI on exercise capacity after TCPC (other types of BCPS patients) | | | | | |
| --- | --- | --- | --- | --- | --- |
| Variables | Peak VO2 | |  | % of normal | |
|  | p-value | Pearson |  | p-value | Pearson |
| **Pre-BCPS** |  |  |  |  |  |
| PAI | 0.273 | 0.442 |  | 0.905 | 0.051* |
| Right PAI | 0.618 | 0.210 |  | 0.648 | 0.193 |
| Left PAI | 0.278 | 0.438 |  | 0.646 | 0.193* |
| Left-to-right ratio | 0.458 | 0.308 |  | 0.489 | 0.288* |
| Symmetry index | 0.449 | 0.314 |  | 0.820 | 0.096 |
| Hemoglobin | 0.081 | 0.649* |  | 0.521 | 0.268* |
| PAP | 0.595 | 0.324* |  | 0.346 | 0.541* |
| TPG | 0.688 | 0.247* |  | 0.409 | 0.483* |
| LAP | 0.341 | 0.389 |  | 0.674 | 0.177* |
| EDP | 0.175 | 0.532 |  | 0.710 | 0.157* |
| SO_2_ | 0.863 | 0.073 |  | 0.993 | 0.004* |
| **Pre-TCPC** |  |  |  |  |  |
| PAI | 0.455 | 0.268* |  | 0.439 | 0.277* |
| Right PAI | 0.050 | 0.632* |  | 0.677 | 0.151* |
| Left PAI | 0.762 | 0.110 |  | 0.493 | 0.246* |
| Left-to-right ratio | 0.203 | 0.440 |  | 0.661 | 0.159* |
| Symmetry index | 0.356 | 0.327 |  | 0.619 | 0.180* |
| Hemoglobin | 0.936 | 0.029 |  | 0.473 | 0.257* |
| PAP | 0.218 | 0.427* |  | 0.476 | 0.256* |
| TPG | 0.636 | 0.171* |  | 0.497 | 0.244* |
| LAP | 0.770 | 0.106* |  | 0.944 | 0.026* |
| EDP | 0.591 | 0.194* |  | 0.361 | 0.324 |
| SO_2_ | 0.965 | 0.016* |  | 0.526 | 0.228 |
| **Post-TCPC** |  |  |  |  |  |
| PAI | 0.742 | 0.174' |  | 0.694 | 0.207 |
| Right PAI | 0.186 | 0.624* |  | 0.840 | 0.107 |
| Left PAI | 0.632 | 0.250 |  | 0.723 | 0.187 |
| Left-to-right ratio | 0.256 | 0.552 |  | 0.656 | 0.233 |
| Symmetry index | 0.053 | 0.806 |  | 0.527 | 0.327 |
| *: negative value |  |  |  |  |  |
