# Supplementary figures and images for: Changes in pulmonary artery size during and after staged extracardiac total cavopulmonary connection
Source: JTCVS Open. 2026 Mar 12;31:101721. doi: 10.1016/j.xjon.2026.101721 (PMC13316346; doi:10.1016/j.xjon.2026.101721)

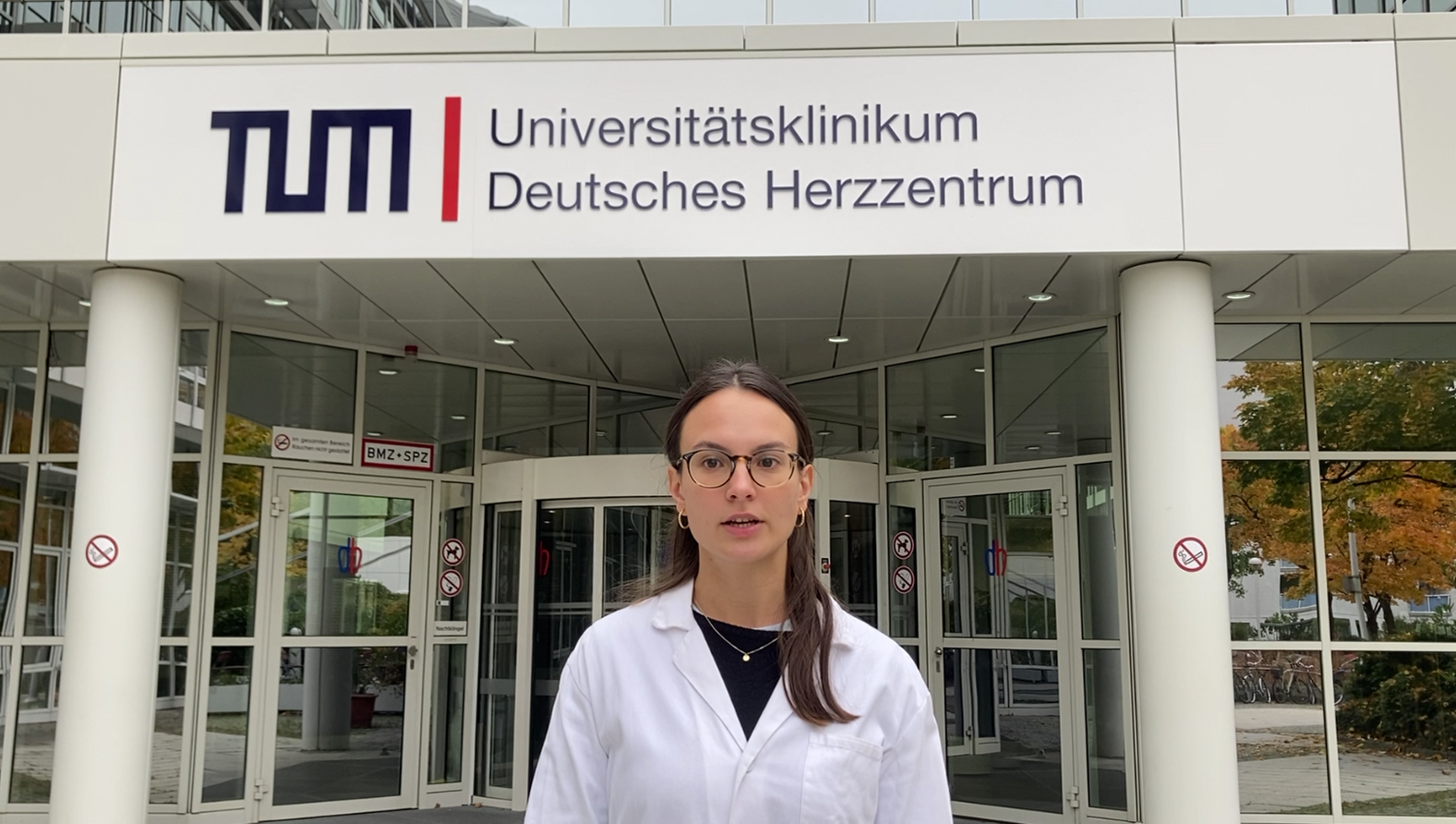

Supplement: Video 1 — The lead author briefly explains the study's importance and relevance. Video available at: https://www.jtcvs.org/article/S2666-2736(26)00144-0/fulltext. [file fx2.jpg]
