# Supplementary material for: Changes in pulmonary artery size during and after staged extracardiac total cavopulmonary connection
Source: JTCVS Open. 2026 Mar 12;31:101721. doi: 10.1016/j.xjon.2026.101721 (PMC13316346; doi:10.1016/j.xjon.2026.101721)
Supplement: Online Data Supplement 2 [file mmc3.docx]

**Supplementary Figure legends**

**Supplementary Figure E1**

PA indices before BCPS regarding type of BCPS. The upper and lower borders of the box indicate the upper and lower quartiles; the middle horizontal line represents the median; and the upper and lower whiskers show the maximum and minimum values in all box-and-whiskers graphs. PA: pulmonary artery, BCPS: bidirectional cavopulmonary shunt.

**Supplementary Figure E2**

PA indices before TCPC regarding type of BCPS. The upper and lower borders of the box indicate the upper and lower quartiles; the middle horizontal line represents the median; and the upper and lower whiskers show the maximum and minimum values in all box-and-whiskers graphs. PA: pulmonary artery, BCPS: bidirectional cavopulmonary shunt, TCPC: total cavopulmonary connection.

**Supplementary Figure E3**

PA indices after TCPC regarding type of BCPS. The upper and lower borders of the box indicate the upper and lower quartiles; the middle horizontal line represents the median; and the upper and lower whiskers show the maximum and minimum values in all box-and-whiskers graphs. PA: pulmonary artery, BCPS: bidirectional cavopulmonary shunt, TCPC: total cavopulmonary connection.

**Supplementary Figure E4**

Changes of PA indices in patients with right-sided BCPS. The upper and lower borders of the box indicate the upper and lower quartiles; the middle horizontal line represents the median; and the upper and lower whiskers show the maximum and minimum values in all box-and-whiskers graphs. PA: pulmonary artery, BCPS: bidirectional cavopulmonary shunt, TCPC: total cavopulmonary connection.

**Supplementary Figure E5**

Changes of PA indices regarding type of BCPS. The upper and lower borders of the box indicate the upper and lower quartiles; the middle horizontal line represents the median; and the upper and lower whiskers show the maximum and minimum values in all box-and-whiskers graphs. PA: pulmonary artery, BCPS: bidirectional cavopulmonary shunt, TCPC: total cavopulmonary connection.
